# Prenatal Exposure to Phthalates, Bisphenols and Their Mixtures and Early Childhood Allergic Conditions and Asthma: Results from the APrON Cohort

**DOI:** 10.3390/ijerph22121875

**Published:** 2025-12-17

**Authors:** Emily Bartram, Gillian England-Mason, Jonathan W. Martin, Amy M. MacDonald, David W. Kinniburgh, Deborah Dewey, Munawar Hussain Soomro

**Affiliations:** 1 Department of Biological Sciences, University of Calgary, Calgary, AB T2N 1N4, Canada; 2 Department of Pediatrics, Cumming School of Medicine, University of Calgary, Calgary, AB T3B 6A8, Canada; 3 Owerko Centre, Alberta Children’s Hospital Research Institute, University of Calgary, Calgary, AB T2N 1N4, Canada; 4 Science for Life Laboratory, Department of Environmental Science, Stockholm University, 114 18 Stockholm, Sweden; 5 Alberta Centre for Toxicology, University of Calgary, Calgary, AB T2N 4N1, Canada; 6 Department of Laboratory Medicine and Pathology, University of Alberta, Edmonton, AB T6G 1C9, Canada; 7 Department of Community Health Sciences, Cumming School of Medicine, University of Calgary, Calgary, AB T2N 4Z6, Canada; 8 Hotchkiss Brain Institute, University of Calgary, Calgary, AB T2N 4N1, Canada

**Keywords:** phthalates, bisphenols, early childhood, allergic conditions, asthma, APrON

## Abstract

Associations between prenatal exposure to phthalates, bisphenols and their mixtures and early childhood allergic conditions and asthma were examined. Five hundred and fifty-six mother–child pairs from the Alberta Pregnancy Outcomes and Nutrition (APrON) cohort participated. Urine samples collected from mothers during the second trimester of pregnancy were analyzed for phthalates and bisphenols. A child health questionnaire, completed by mothers when children were 12, 24, and 36 months, asked whether children had experienced allergic conditions (i.e., food allergies, eczema, rash) or asthma. In single-chemical models, associations varied with child age. Higher prenatal concentrations of mono-benzyl phthalate (MBzP) were associated with lower odds of eczema at 12 months. At 36 months, higher mono-methyl phthalate (MMP) was associated with increased odds of eczema, whereas higher mono-carboxy-octyl phthalate (MCOP) was associated with reduced odds. Higher prenatal MCOP was also associated with higher odds of rash at 12 months, and higher MMP was associated with higher odds of rash at 36 months. Higher bisphenol S (BPS) was associated with increased odds of asthma at 12 months but decreased odds of eczema and rash at 36 months. Sex-specific effects were also noted. In multi-chemical exposure least absolute shrinkage and selection operator (LASSO) models, several phthalate metabolites and BPS were selected as the best predictors of eczema and rash at 36 months of age. Bayesian kernel machine regression (BKMR) mixture models suggested that BPS was the most important chemical in predicting eczema in children at 36 months, while MMP and BPS were the most important chemicals in predicting rash at 36 months. Prenatal exposure to certain phthalate metabolites and BPS predicted allergic conditions and asthma in young children, with patterns varying by age and sex. Prenatal exposure to these chemicals may differentially influence immune development and contribute to the development of early-life allergic conditions, with potentially sex-specific susceptibility.

## 1. Introduction

Exposure to endocrine-disrupting chemicals (EDCs) such as phthalates and bisphenols is linked with increased risk of allergic conditions and asthma in children [1]. Phthalates are a group of chemicals commonly used as plasticizers in building materials, cosmetics, personal care products, medical equipment, toys and food packaging because of their flexibility, durability and resistant kinking properties [2]. Bisphenols serve as foundational building blocks to create hard, clear polycarbonate plastics and durable epoxy resins used in food and drink can linings, water bottles, electronics, and dental sealants [3]. These chemicals are ubiquitous in the environment and can leach from products into food, water, and dust, resulting in routine exposure through inhalation, ingestion, and dermal absorption through the skin [4,5].

A growing body of research suggests that EDCs may contribute to the increasing prevalence of allergic conditions and asthma in young children [1,6,7,8]. Both phthalates and bisphenols can cross the placental barrier and disrupt fetal endocrine development [9,10,11]. This could have consequences for integumentary and respiratory systems, including congestion and wheezing, shortness of breath, inflammation, and hives, redness, and itchiness, which are characteristics of allergies and asthma [12]. Over the past 30 years, the prevalence of these conditions has increased substantially in Canada. In Alberta, asthma prevalence increased from 3.9% to 12.3% in females and from 3.5% to 11.6% in males between 1995 and 2015 [13]. National data shows increases in self-reported allergies from 7.1% to 9.3% between 2010 and 2016 [14], and clinical data from Ontario indicate that the prevalence of eczema is greater among children (9.9%) than adults (1.8%) [15]. Furthermore, the overall prevalence of pediatric atopic dermatitis in Canada has been estimated to be 15.1% [16]. EDC exposure has been proposed as a potential contributor to these rising trends [17].

EDCs can influence immune and inflammatory pathways. Mechanistic evidence suggests that bisphenols can interact with estrogen receptors, triggering hormonal imbalances and pro-inflammatory signalling. Bisphenol A (BPA) can bind to peroxisome proliferator-activated receptors (PPARs) and toll-like receptors, disrupting the balance between immune cells, namely type 1 helper (Th1) cells, which are part of the pro-inflammatory pathways, and type 2 helper (Th2) cells, which regulate the anti-inflammatory response [18]. Prenatal phthalate exposure has been associated with reductions in regulatory T (Treg) cells, which are critical in immune response modulation [19] and may induce epigenetic changes, such as altered DNA methylation [20], which increase susceptibility to allergic disease and asthma later in life [21]. These findings support the hypothesis that prenatal exposure to EDCs may contribute to developmental programming of immune function and influence allergic and asthma outcomes across the lifespan [22,23].

Although previous epidemiological studies have examined associations between prenatal exposure to phthalates or bisphenols and childhood allergic and asthma outcomes, results remain inconsistent and often vary by child sex and timing of exposure assessment [24,25,26,27,28]. Few studies have evaluated which specific EDCs serve as the strongest predictors of these outcomes or considered the combined effects of EDC mixtures during fetal development [25,29,30].

To address these gaps, we examined associations between prenatal exposure to individual phthalates metabolites and BPA and Bisphenol S (BPS), as well as mixture effects of these targeted chemicals, and the development of allergic conditions and asthma in early childhood. Using data from a Canadian pregnancy cohort, we used multiple logistic regression analyses to examine the associations between the individual bisphenols and phthalate metabolites and each allergic and asthma disease outcome. We then applied least absolute shrinkage and selection operator (LASSO) regression to identify the best predictors of these associations [31] and Bayesian kernel machine regression (BKMR) to assess mixture effects of phthalates and bisphenols [32].

## 2. Methods

### 2.1. Study Population and Data Collection

This study drew on a subsample of maternal-child pairs (N = 556) from the Alberta Pregnancy Outcomes and Nutrition (APrON) cohort [33,34]. Participants were eligible if (1) the mother provided a second trimester urine sample that was quantified for BPA, BPS, and phthalate metabolites and (2) a child health questionnaire was completed by the mother at 12, 24, or 36 months of age.

### 2.2. Ethical Approval

This research was approved by the Conjoint Health Research Ethics Board at the University of Calgary, Canada, and the University of Alberta Health Research Biomedical Panel, Edmonton, Canada. Written informed consent was provided by all the women prior to the completion of questionnaires and urine sample collection.

### 2.3. Exposure Assessment

#### 2.3.1. Phthalates

Maternal urine samples collected in the second trimester of pregnancy (average gestational age = 17 weeks, standard deviation = 2.1) were analyzed at the Alberta Centre for Toxicology, University of Calgary for phthalate metabolites using previously described analytical methods [35,36]. Fourteen metabolites were measured: mono(2-ethylhexyl) phthalate (MEHP), mono(2-ethyl-5-hydroxy-hexyl) phthalate (MEHHP), mono(2-ethyl-5-oxohexyl) phthalate (MEOHP), mono(2-ethyl-5-carboxypentyl) phthalate (MECPP), mono-benzyl phthalate (MBzP), mono-carboxy-octyl phthalate (MCOP), mono-carboxy-isononyl phthalate (MCNP), and mono-isononyl phthalate (MNP), mono-n-butyl phthalate (MBP), mono-iso-butyl phthalate (MiBP), mono-ethyl phthalate (MEP), mono-methyl phthalate (MMP), mono-cyclohexyl phthalate (MCHP), and mono-n-octyl phthalate (MOP). MCHP and MOP were excluded from the analyses due to low detection frequency (<50%).

The di (2-ethylhexyl) phthalates (DEHP), MECPP, MEHHP, MEOHP and MEHP were summed to obtain the molar concentration of ΣDEHP. The molar sum (nmol/mL) was calculated by dividing each metabolite concentration by its molecular weight and summing the resulting values.

Metabolites were quantified using high-performance liquid chromatography-tandem mass spectrometry (HPLC-MS/MS) (QTRAP 5500, AB Sciex, Concord, ON, Canada) operating in negative multiple reaction monitoring (MRM) mode. An Agilent 1200 HPLC system (Agilent Technologies, LabX, Mississauga, ON, Canada), was used to separate the metabolites on a 100 Å ~ 2.1 mm BetaSil Phenyl Column (Thermo Scientific, Burlington, ON, Canada) with a 10 μL injection volume and a constant column temperature of 40 °C. Metabolites were identified based on two MRM transitions at the expected retention time. The limit of detection (LOD) for all phthalate metabolites was 0.10 μg/L. Consistent with recommended practice, values below the LOD were assigned the value of LOD/√2 [37].

#### 2.3.2. Bisphenols

Second trimester maternal urine samples (mean gestational age = 17 weeks, standard deviation = 2.1 weeks) were analyzed for total (conjugated + free) bisphenol concentrations using previously described methods [35,38,39]. Briefly, metabolites were deconjugated by incubation with a mixture of β-glucuronidase and sulfatase. Total bisphenols were quantified using online solid-phase extraction coupled to HPLC-MS/MS and an Orbitrap Elite hybrid mass spectrometer (Thermo Fisher Scientific, Waltham, MA, USA). The LODs were 0.32 µg/mL for BPA and 0.10 µg/mL for BPS. Concentrations below the LOD were assigned values of LOD/√2 [37].

### 2.4. Outcome Assessment: Food Allergy, Eczema, Rash, and Asthma

Mothers completed an adapted version of the Child Health Questionnaire, a validated measure [40], when children were 12, 24, and 36 months of age. The questionnaire assessed common health conditions during the preceding 12 months, including food allergies, skin problems (e.g., rashes, eczema), and respiratory symptoms indicative of asthma (e.g., wheezing or whistling in the chest). Mothers reported whether their child had experienced each condition (yes/no) and could provide additional descriptive information. Child health outcomes were based exclusively on maternal report; no clinical assessments or medical records were available to validate parent-reported outcomes. Food allergy reports were interpreted according to the criteria for oral allergy syndrome [41,42,43]. Skin conditions, were coded as present if mothers reported eczema or dermatitis, including parent-reported diagnoses [42]. Reactions to irritants such as bug bites, chlorine or soaps were excluded due to their non-specific nature. Information on skin conditions was available only at the 12- and 36-month assessments. Asthma was coded as present if mothers reported asthma, use of a puffer, wheezing, difficult breathing or chronic cough [44].

### 2.5. Covariates

Potential confounders were identified a priori based on previous literature [8,24,25,45,46,47]. Maternal sociodemographic and biological covariates included educational attainment (university degree/postgraduate, high school/technical/trade school), marital status (married or cohabiting, single, divorced, separated or widowed), parity (0, 1, 2), household income (<$70,000, ≥$70,000), age at delivery (<25 years, 25–29 years, 30–34 years, ≥35 years), pre-pregnancy body mass index (BMI, <18.5, 18.5–24.9, 25.0–30.0, >30 kg/m^2^). Child covariates included gestational age at birth (<37 gestational week, ≥37 gestational week), child sex (male, female). Maternal self-reported race was categorized as white or non-white. Urinary creatinine concentration was included to account for dilution variability in urinary phthalate metabolite and bisphenol measurements.

### 2.6. Statistical Analysis

Concentrations of phthalate metabolites and bisphenols were natural log (ln) transformed to reduce the influence of extreme values and to improve interpretability, an approach commonly used in environmental exposure analyses [48]. We summarized sociodemographic characteristics using means and standard deviations (SDs) for continuous variables, and frequencies and proportions for categorical variables. The prevalence and incidence of each health outcome was calculated.

Multiple logistic regression models were used to estimate the associations between the individual phthalate metabolites and bisphenols (i.e., single exposure models) and each allergic and asthma-related outcome. We calculated crude, adjusted, and sex-stratified odds ratios with 95% confidence intervals (CI). Models were adjusted for maternal education, maternal age, pre-pregnancy BMI, marital status, race, household income, parity, child sex, gestational age at birth, and creatinine. Statistical significance was defined as α = 0.05; borderline significance was identified as α = 0.10. To address multiple testing, we applied the Benjamini–Hochberg procedure to control for false discovery rate (FDR) at *p* < 0.05 [49].

Because birth weight has been associated with early childhood allergic conditions and asthma [50,51,52], we performed a sensitivity analysis excluding infants with low birth weight (<2500 g) and high birth weight (>4500 g).

To identify the chemical metabolites most predictive of each outcome, we first applied LASSO penalized regression [31,53,54] to all exposures (i.e., 12 phthalates, 2 bisphenols). LASSO simultaneously performs variable selection and shrinkage [31,55], which reduces the risk of overfitting and improves interpretability by yielding sparse models with more precise coefficient estimates [31]. Ten-fold cross-validation was used to select the optimal values of lambda (λ) corresponding with the minimum mean squared error (MSE) as recommended [56]. Metabolites with coefficients not shrunk to zero were retained as predictors. We then applied double LASSO logistic regression [57,58], including all 14 chemical exposures. Double LASSO corrects for LASSO’s shrinkage bias (if any) [58,59], improves predictive accuracy, and avoids over-selection of spurious predictors relative to linear, logistic, polynomial, or ridge regression [31,60]. It also reduces error as it does not over-select potentially spurious covariates, which increases the statistical power to identify the chemicals most predictive of allergic and asthma outcomes [57,58]. Statistical significance was defined as α = 0.05 and 95% confidence intervals (CI) were reported; for these analyses, borderline significance was identified as α = 0.10. Statistical analyses were conducted in SPSS Version 28 (IBM Corp, Armonk, NY, USA, 2019) and Stata Version 19.5 (Stata Corp LP, 4905 Lakeway Drive, College Station, TX 77845, USA).

We next evaluated mixture effects using BKMR, a non-parametric Bayesian variable selection method using the bkmr R package (version 4.4.0). BKMR was implemented using a probit link to accommodate binary outcomes (e.g., eczema vs. no eczema) using 20,000 Markov Chain Monte Carlo iterations [61]. Exposures were grouped into group 1, phthalate metabolites (i.e., MMP, MEP, MBP, MiBP, MECPP, MEHHP, MEOHP, MEHP, MBzP, MCOP, MNP, MCNP) and group 2 bisphenols (i.e., BPA, BPS). For each group, we estimated posterior inclusion probabilities (group PIPs). Conditional PIPs were then calculated for the individual chemicals within each group. PIPs values range from 0 to 1, and a threshold of ≥0.5 was used to identify the groups or chemicals with meaningful inclusion probabilities [62,63].

## 3. Results

### 3.1. Study Population Characteristics

Mother–child pairs (N = 556) were primarily white (86.2%). Average maternal age at delivery was 32 years (SD ± 3.94; range = 16.6 to 42.8 years). Most participants had a university education (72.8%) and were married or cohabiting with a partner (96.2%). Over half (55.4%) of the participants were first time mothers, with an average pre-pregnancy BMI of 24.7 (SD ± 5.1) kg/m^2^. Most of the children were born at or after 37 weeks’ gestation (92.6%), with an average birth weight of 3364.9 g (SD ± 538.6 g), and 51.4% were males (Table 1). Significant differences were observed between the study subsample and the original APrON cohort for some sociodemographic characteristics. The sociodemographic characteristics of the study population by sex of the child are shown in Table 2.

### 3.2. Maternal Chemical Exposures

Detection rates and distributions of the phthalate metabolites and bisphenols are presented in Table 3. Detection rates exceeded 98% for all phthalate metabolites, except for MCNP, which had a detection rate of 91%. Among the metabolites, MEP showed highest geometric mean urinary concentration (50.4 µg/L), whereas MCHP and MOP had the lowest geometric means (0.1 µg/L). For bisphenols, the detection rate was 92.3% for BPA and 58.6% for BPS.

Spearman correlation coefficients for ln-transformed urinary concentrations of phthalate metabolites and bisphenols are shown in Table S1. Overall, metabolites were positively correlated, with coefficients ranging from ρ = 0.14 to 0.98. As expected, the DEHP metabolites (i.e., MEOHP, MEHHP, MECPP, MEHP) were strongly intercorrelated (ρ ≥ 0.80 to 0.98, *p* < 0.05). The correlations between bisphenols and phthalate metabolites ranged from ρ = 0.18, *p* < 0.05 to ρ = 0.50, *p* < 0.05.

### 3.3. Prevalence and Incidence of Food Allergy, Eczema, Rash, and Asthma

The prevalence of food allergies was 7.2%, 18.5% and 28.1% at 12, 24, and 36 months, respectively. Eczema prevalence was 18.9% at 12 months and increased to 35.4% at 36 months, while rash prevalence rose from 33.9% at 12 months to 61.1% at 36 months. Asthma prevalence was low but increased from 0.4% at 12 months to 1.9% at 24 months and 5.7% at 36 months (see Table 4).

Incidence patterns differed somewhat from prevalence. The incidence of food allergies increased from 7.2% at 12 months to 11.3% at 24 months but declined to 9.5% at 36 months. Incidence of eczema decreased from 18.9% at 12 months to 16.5% at 36 months, and rash incidence declined from 33.9% at 12 months to 27.1% at 36 months. In contrast, the incidence of asthma steadily increased with age from 0.4% at 12 months to 1.6% at 24 months and 3.9% at 36 months (see Table 4).

### 3.4. Associations Between Single Chemical Exposures and Maternal Reported Food Allergy, Eczema, Rash, and Asthma

In adjusted logistic regression models, prenatal phthalate exposures were not associated with food allergies at any age (Table 5). Higher maternal MBzP concentrations were associated with lower odds of eczema at 12 months (AOR = 0.76, 95% CI = 0.60–0.97, *p* = 0.03) (Table 5). At 36 months, higher maternal MMP was associated with increased odds of eczema (AOR = 1.29, 95% CI = 1.00–1.68, *p* = 0.04), whereas MCOP was associated with reduced odds (AOR = 0.78, 95% CI = 0.64–0.96, *p* = 0.02). Higher prenatal MCOP was also associated with higher odds of rash at 12 months (AOR = 1.21, 95% CI = 1.03–1.41, *p* = 0.01), and higher MMP was associated with higher odds of rash at 36 months (AOR = 1.35, 95% CI = 1.08–1.69, *p* = 0.008). No associations were observed between prenatal phthalates and asthma.

For bisphenols, higher prenatal BPS concentrations showed a trend for increased odds of asthma at 12 months (AOR 2.15, 95% CI = 0.88–5.23, *p* = 0.08), but reduced odds of eczema (AOR = 0.72, 95% CI = 0.56–0.93, *p* = 0.01) and rash (AOR = 0.81, 95% CI = 0.67–0.98, *p* = 0.03) at 36 months. Prenatal BPA concentrations were not associated with any outcomes (Table 6).

After applying the Benjamini–Hochberg procedure for correction for multiple comparisons, none of the above associations remained statistically significant (adjusted *p*-value > 0.05).

### 3.5. Sex-Stratified Associations Between Single Chemical Exposures and Maternal Reported Food Allergy, Eczema, Rash, and Asthma

Among females, associations between prenatal phthalate exposure and allergic or asthma outcomes varied by age. At 24 months, prenatal MMP was associated with lower odds of food allergies (AOR = 0.58, 95% CI = 0.36–0.93, *p* = 0.02), whereas at 36 months, MMP was associated with higher odds of food allergies (AOR = 1.60, 95% CI = 1.06–2.42, *p* = 0.02). Higher prenatal MEP (AOR = 0.69, 95% CI = 0.52–0.92, *p* = 0.01) and MiBP (AOR = 0.62, 95% CI = 0.41–0.95, *p* = 0.02) were associated with lower odds of eczema at 12 months; however, higher prenatal MCNP (AOR =1.25, 95% CI = 1.05–1.50, *p* = 0.01) and MBzP (AOR = 1.30, 95% CI = 1.00–1.69, *p* = 0.04) were associated with increased odds of eczema. For rash, higher MEP (AOR = 0.77, 95% CI = 0.61–0.95, *p* = 0.02) was protective at 12 months. At 36 months, higher MCOP (AOR = 0.23, 95% CI = 0.06–0.90, *p* = 0.03) and MNP (AOR =0.38, 95% CI = 0.15–0.91, *p* = 0.03) were associated with lower odds of asthma (see Table S2).

In males, higher MBzP was associated with lower odds of rash (OR = 0.72, 95% CI = 0.55–0.96, *p* = 0.02) and eczema (AOR = 0.66, 95% CI = 0.46–0.92, *p* = 0.01) at 12 months, whereas higher MCOP was associated with higher odds of rash at the same age (AOR =1.34, 95% CI = 1.08–1.66, *p* = 0.007). At 36 months, higher prenatal MMP was associated with increased odds of eczema (OR = 1.47, 95% CI = 1.02–2.12, *p* = 0.03) and rash (OR =1.45, 95% CI = 1.06–1.98, *p* = 0.02) (see Table S2).

For bisphenols, higher prenatal BPS was associated with lower odds of food allergy at 24 months in females (AOR = 0.64, 95% CI = 0.39–1.04, *p* = 0.07) and at 36 months in males (AOR = 0.62, 95% CI = 0.36–0.98, *p* = 0.04) (see Table S3). A trend association was noted between BPS and eczema in females at 36 months (AOR = 0.70, 95% CI = 0.48–1.02, *p* = 0.06). In males, BPS displayed a trend association with asthma at 12 months (AOR = 3.24, 95% CI = 0.97–10.01, *p* = 0.05). BPA was not significantly associated with eczema, rash, or asthma in either males or females at any age.

After applying the Benjamini–Hochberg procedure for correction for multiple comparisons, none of the above associations remained statistically significant (adjusted *p*-value > 0.05).

### 3.6. Sensitivity Analysis

In a sensitivity analysis excluding infants with low and high birthweight (N = 46), all statistically significant associations in the adjusted logistic regression models remained significant, supporting the robustness of main findings.

### 3.7. Multiple Chemical Exposure Models

In the LASSO model for eczema at 36 months, MMP, MEP, MEHP, MECPP, MCOP, MCNP and BPS were selected as the best predictors, yielding an MSE of 0.0162 (Figure S1A). In the subsequent double LASSO logistic regression that included all phthalate metabolites and bisphenols, MMP was positively associated with eczema (AOR: 1.42, 95% CI: 1.07–1.88, *p* = 0.01), whereas MCOP (AOR: 0.67, 95% CI: 0.51–0.89, *p* = 0.006) and BPS (AOR: 0.73, 95% CI: 0.57–0.93, *p* = 0.01) were inversely associated (Table 7).

For rash at 36 months of age, the LASSO model selected MMP, MEHP, MEOHP, MCOP and BPS as the best predictors (MSE log λ = 0.0219; Figure S1B). The double LASSO logistic regression revealed positive associations for MMP (AOR: 1.41, 95% CI: 1.11–1.81, *p* = 0.005) and MEHP (AOR: 1.49, 95% CI: 1.11–2.00, *p* = 0.007), and inverse associations for MCOP (AOR: 0.82, 95% CI: 0.69–0.98, *p* = 0.03) and BPS (AOR: 0.79, 95% CI: 0.66–0.96, *p* = 0.01) (Table 7).

As LASSO models identified predictors of eczema and rash at 36 months, we conducted BKMR analyses to evaluate the joint and chemical group effects of phthalate metabolites and bisphenols on these outcomes. Tables S4 and S5 summarize the hierarchical variable selection results for group and conditional PIPs for eczema and rash, respectively

For eczema at 36 months, bisphenols demonstrated the highest group-level PIP (0.69), indicating a strong contribution of this chemical class, followed by phthalates with a moderate group PIP of 0.47. For rash at 36 months, both bisphenols and phthalates showed high group-level importance (bisphenols: PIP = 0.65; phthalates: PIP = 0.53; Table S5). Conditional PIPs within each chemical class were generally low (<0.20), but BPS consistently exhibited high conditional importance for both eczema (conditional PIP = 0.87) and rash (conditional PIP = 0.88). A moderate conditional PIP was observed for MMP in relation to rash (conditional PIP = 0.47) (Table S5).

Sex-stratified BKMR model showed similar patterns. Among females, phthalates exhibited a group PIP of 0.64 for eczema, followed by bisphenols (group PIP = 0.49; Table S6). Among males, bisphenols had a group PIP of 0.68 for rash, while phthalates showed a lower group PIP of 0.40 (Table S7). Consistent with the overall models, BPS showed high conditional PIPs in both sex-stratified models, 0.72 for eczema in females and 0.80 for rash in males. MMP again showed a moderate conditional PIP for rash in males (conditional PIP = 0.30) (Table S7).

Univariate exposure–response functions demonstrated mostly weak associations across overall and sex-stratified BKMR models (Figures S2–S5). Each graph depicts the association between one natural log-transformed analyte and the outcome while holding all other exposures at their median values. The posterior mean estimates (blue lines) were generally close to the null, and 95% confidence intervals (grey shaded areas) were narrow to moderately wide, indicating varying but generally limited precision.

**Figure 1:**
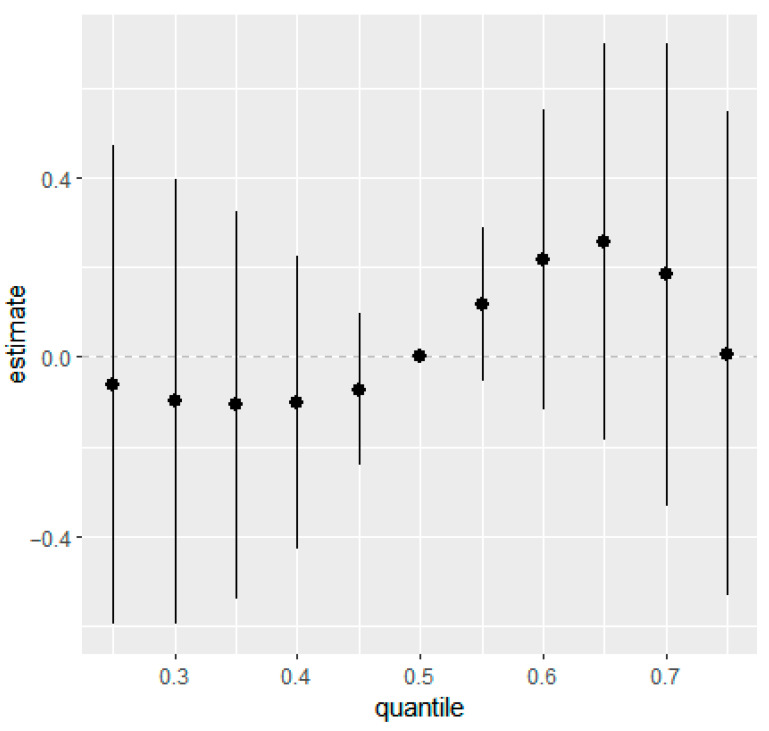
Overall mixture effect (95% CIs) of prenatal phthalate and bisphenol exposure on eczema at 36 months of age estimated using BKMR. This plot illustrates the change in predicted probability of eczema when all ln-transformed analytes were set simultaneously to a given quantile for their distributions compared to when they are held at their median values. Adjusted for maternal education, maternal age, maternal pre-pregnancy BMI, marital status, gestational age at birth, household income, parity, child sex, race, and creatinine.

**Figure 2:**
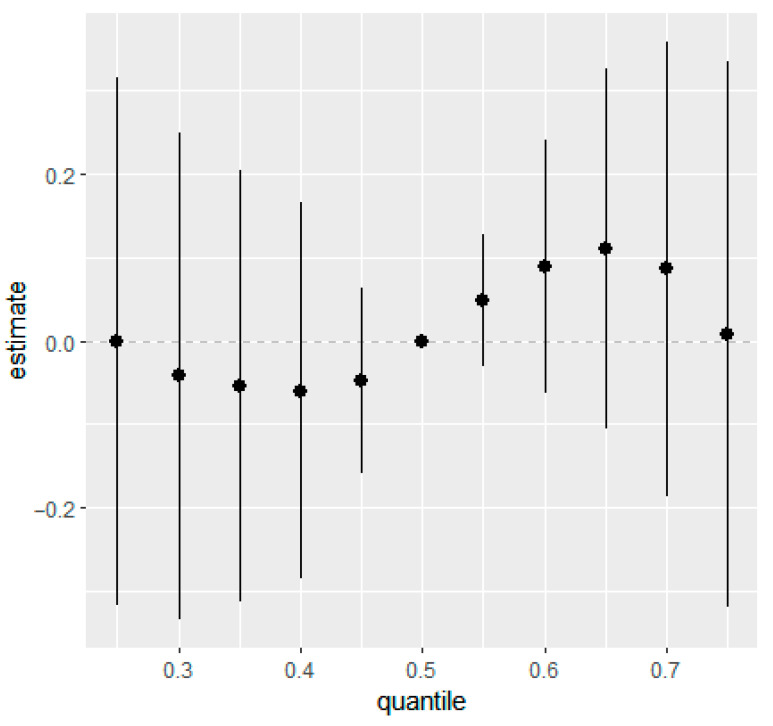
Overall mixture effect (95% CIs) of prenatal phthalate and bisphenol exposures on rash in children at 36 months of age estimated from BKMR. This plot illustrates the change in predicted probability of rash when all ln-transformed analytes were set simultaneously to a given quantile for their distributions compared to when they are held at their median values. Adjusted for maternal education, maternal age, maternal pre-pregnancy BMI, marital status, gestational age at birth, household income, parity, child sex, race, and creatinine.

Similarly, overall mixture response functions showed week associations for eczema and rash at 36 months in the combined (Figure 1 and Figure 2) and the sex-stratified models (Figures S6 and S7). Posterior mean estimates of the change in outcome risk across quantiles of joint exposure mixture were close to zero at lower quantiles and displayed increasing trends only at higher quantiles (≥0.6). However, the 95% CIs were wide and generally overlapped zero, indicating substantial uncertainty and no statistically robust evidence of associations between higher prenatal mixture exposures and increased eczema or rash risk.

## 4. Discussion

In this prospective birth cohort, we found evidence that prenatal exposure to phthalate metabolites and BPS was associated with allergic conditions and asthma in early childhood. In single chemical models, higher prenatal concentrations of several phthalate metabolites (MMP, MBzP, MCOP) and BPS were associated with increased odds of eczema and rash at 36 months, and asthma at 12 months; however, these associations were attenuated after applying the Benjamini–Hochberg procedure for correction for multiple comparisons. We also observed several sex-specific relationships. Among females, MMP, MEP and MiBP were associated with increased odds of food allergies at 24 months and lower odds of eczema and rash at 12 months, whereas MCNP and MBzP were linked to higher odds of eczema at 36 months. In addition, MCOP and MNP were associated with lower odds of asthma in females at 36 months. In males, MMP predicted higher odds of eczema and rash at 36 months of age, while MBzP was associated with lower odds of these outcomes at 12 months. Again, these findings were attenuated after correction for multiple comparisons. Findings from LASSO multi-chemical exposure models were consistent with single chemical analyses. In BKMR mixture models, BPS was the most influential contributor to eczema at 36 months, while MMP and BPS contributed most strongly to rash at the same age. Taken together, these results suggest that prenatal exposure to BPS and specific phthalate metabolites may differentially influence immune development and contribute to early life allergic conditions, with potential sex-specific susceptibility.

Epidemiological evidence supports links between prenatal exposure to phthalates and bisphenols and altered immune function, allergic conditions and asthma in children [24,25,26]. Prenatal exposure to phthalates has been associated with both early onset (0–24 months) and late onset (24–60 months) of eczema [8], and trimester-specific exposure has been linked to wheeze and asthma in males aged four to six years, highlighting potential sex-specific effects [26]. Findings for prenatal BPA exposure have been mixed, with some studies reporting no associations [25], and others reporting increased sex-specific risks of asthma, wheeze, eczema and food allergy [24,27,28]. Research assessing BPS is limited, but recent findings indicated that pregnancy averaged BPS was associated with increased odds of asthma in males [28].

Studies from the REPRO_PL cohort suggest that specific phthalate metabolites may influence allergic outcomes differently across developmental stages. For example, higher maternal MBzP was associated with increased risk of food allergy in children during the first two years of life [46], while higher MEHP predicted increased the risk at 9 years of age [64]. We did not observe associations between phthalate or bisphenol exposures and food allergy in the first three years of life.

Several international birth cohort studies from the USA, France, Greenland, and Ukraine have reported associations between phthalate metabolites (i.e., MBzP, MiBP, MCOP) and childhood eczema [8,65,66]. Cross-sectional evidence from South Korea similarly reported associations between MEP, MBzP, MCOP, MCNP, and DEHP metabolites, and atopic dermatitis in children [67]. Consistent with this literature, we observed a positive association between MMP and eczema at 36 months of age, an inverse association with MCOP at the same age, and a positive association with MBzP at 12 months. We also identified positive associations between MCOP and MMP and rash at 12 and 36 months. Contrary to prior studies reporting associations between prenatal exposure to BPA and eczema [45,68], we did not find evidence for BPA; instead, we observed an inverse association between BPS and eczema at 36 months.

Previous work has also identified MCOP as a predictor of asthma and reduced lung function at age seven [25], whereas our findings indicate that MCOP and MNP were protective against asthma in girls at 36 months. Biological mechanisms underlying these divergent findings remain unclear, but phthalates are known to interact with multiple immune pathways, suggesting the potential for both protective and adverse effects depending on dose, timing, and immunologic context. Research suggests that phthalates may alter cytokine production, reduce regulatory T-cell populations, and exert both pro- and anti-inflammatory effects [19,69,70], potentially leading to complex and chemical-specific patterns of immune disruption.

The concentrations of phthalate metabolites and bisphenols observed in the APrON cohort were consistent with levels reported in other North American populations [71] and are generally considered moderate on a global scale [64,65]. The concentrations were also comparable to those in European cohorts [8,46,72,73] but lower than levels reported in studies from China [2,54], likely reflecting differences in regulatory policies and industrial practices that affect the consumer products used by the general public, and community and workplace exposures.

Emerging evidence suggests that low-level exposure to certain phthalates may elicit a mild, adaptive immune response without triggering substantial inflammation; this could promote immune tolerance and potentially protect against allergic asthma in some cases [26,74]. For example, higher phthalate exposure in healthy adults has been associated with IL-6 levels, a cytokine typically involved in pro-inflammatory responses [69]. Similarly, recent work has shown that higher third-trimester exposure to phthalates such as DMP, DnBP, and DiBP was associated with decreased cord blood immune cell populations (e.g., IL-1β, IL-9, Th2, Treg), whereas another phthalate (i.e., DEP) was positively associated with the immune marker TNF-α [70]. Prenatal exposure to phthalates and bisphenols may also contribute to the development of allergic diseases and asthma by disrupting endocrine and immune pathways [75]. Phthalates can interfere with cytokine and chemokine production [76,77], reduce Treg cell levels [19], and potentially impair immune suppression necessary to prevent atopic dermatitis [78,79]. BPA may promote the production of IL-4 and other Th2-related cytokines, which may heighten allergic susceptibility [80]. Additionally, allergic disease expression may vary across childhood with changes in immune maturation, physical activity, environmental exposures, and metabolism [8,81]. The current body of evidence suggests that phthalate metabolites, BPA and BPS may influence immune pathways in distinct ways, which could contribute to both protective and adverse effects on the development of allergic disease. Further research is needed to elucidate underlying mechanisms and to determine how prenatal exposure to phthalates, bisphenols, and other EDCs contributes to allergic conditions and asthma in childhood.

Relatively few studies have examined mixtures of EDCs in relation to allergic conditions and asthma. CHAMACOS findings identified MCOP as a key contributor to asthma in BKMR models [25], while results from the Hokkaido cohort differed depending on analytic method, with MINP and MEOHP weighted heavily in weighted quantile sums (WQS) models and DINP and DEHP highlighted in BKMR model [30]. A case–control study from Taiwan found that MBzP, MiBP, and MiNP contributed most strongly to asthma in WQS analyses [29]. Our findings add to this emerging body of work by identifying MMP, MCOP and BPS as the most influential predictors of eczema and rash in LASSO models, and BPS and MMP as key contributors to eczema and rash in BKMR models at 36 months. Together, the mixed findings of these mixture analyses underscore the need for further investigations using multiple mixture analysis approaches to clarify the importance of individual chemicals in chemical mixtures.

This study has several strengths, including its prospective design and the availability of extensive prenatal and early-life covariates within the APrON cohort. The use of logistic regression, LASSO, double LASSO and BKMR enabled rigorous evaluation of both single-chemical and mixture effects. However, several limitations warrant consideration. Exposure assessment was based on a single second trimester urine sample, which may not fully capture exposure variability across pregnancy. Postnatal exposure was not measured, limiting our ability to examine combined prenatal and postnatal effects. Breast feeding data were incomplete, precluding evaluation of potential effect modification. Asthma outcomes were broadly defined, potentially inflating prevalence estimates and misclassification. The cohorts’ relatively high socioeconomic status and lack of clinical verification of allergic outcomes may limit generalizability. Finally, none of the overall associations in single chemical models remained statistically significant after correction for multiple comparison, raising the possibility of false positives and emphasizing the need for cautious interpretation.

## 5. Conclusions

Prenatal exposure to certain phthalate metabolites and BPS may be associated with allergic conditions and asthma in young children, with patterns varying by age and sex. Single-chemical models identified MMP, MBzP, MCOP and BPS as predictors of eczema, rash, and asthma, while mixture analyses highlighted BPS and MMP as the most influential contributors to eczema and rash at 36 months. These findings suggest that BPS and selected phthalate metabolites may disrupt early-life immune development and contribute to allergic outcomes. Future research should incorporate repeated prenatal measurement of exposures and postnatal exposure data and examine sex-specific effects to clarify exposure timing, susceptibility windows, and the influence of sex on the development of allergic conditions and asthma in children.

## Figures and Tables

**Table 1:** Sociodemographic characteristics of the APrON subsample included in the present study and the overall APrON cohort.

Characteristics	Present Study (N = 556)		APrON Cohort (N = 2189)		
	N	%	N	%	*p* Value
**Maternal Age**					
<25	18	3.2	148	6.8	0.005
25–29	147	26.4	586	26.7	
30–34	262	47.2	912	41.7	
≥35	18	3.2	148	6.8	
Missing	7	1.3	79	3.6	
**Parity**					
0	304	54.7	1194	54.6	0.19
1	196	35.3	717	32.7	
≥2	49	8.8	223	10.2	
Missing	7	1.3	55	2.5	
**Pre-Pregnancy BMI**					
<18.5	16	2.9	79	3.6	0.83
18.5–24.9	343	61.7	1339	61.2	
25–30	126	22.6	482	22.0	
>30	71	12.8	289	13.2	
Missing	2	0.4	79	3.6	
**Maternal Education**					
Less than high school/high school diploma/technical/ trade school	141	25.4	663	30.3	0.01
University degree	405	72.8	1459	66.6	
Missing	10	1.8	67	3.1	
**Smoking during pregnancy**					
Yes	6	1.1	47	2.2	0.23
No	531	95.5	2058	94.0	
Missing	19	3.4	84	3.8	
**Gestational age**					
<37 gestational week	34	6.1	197	9.0	0.0001
≥37 gestational week	515	92.6	1832	83.7	
Missing	7	1.3	160	7.3	
**Household Income**					
<$70,000	100	18.0	469	21.2	0.0001
≥$70,000	447	80.4	1608	73.6	
Missing	9	1.7	112	5.2	
**Marital Status**					
Married/cohabitating	535	96.2	1998	91.3	0.0004
Unmarried/single/ divorce/separated/ widowed	14	2.6	115	5.2	
Missing	7	1.3	76	3.5	
**Race**					
White	479	86.2	1774	81.0	0.0008
Non-white	76	13.7	343	15.7	
Missing	1	0.2	72	3.3	
**Child birthweight (g)**					
<2500	28	5.0	114	5.2	0.05
≥2500	521	93.7	2006	91.6	
Missing	7	1.3	69	3.2	
**Child Sex**					
Female	263	47.3	1003	45.8	0.10
Male	286	51.4	1124	51.4	
Missing	7	1.3	62	2.8	

BMI = body mass index.

**Table 2:** Sociodemographic characteristics of the APrON subsample included in the present study by sex of the child (N = 549; 7 missing information on child sex).

Characteristics	Females (N = 263)		Males (N = 286)	
	N	%	N	%
**Maternal Age**				
<25	7	2.7	10	3.5
25–29	63	24.0	82	28.7
30–34	131	49.8	129	45.1
≥35	62	23.6	65	22.7
**Parity**				
0	133	50.6	171	59.8
1	113	43.0	83	29.0
≥2	17	6.5	32	11.2
**Pre-Pregnancy BMI**				
<18.5	8	3.0	6	2.1
18.5–24.9	161	61.2	178	62.2
25–30	62	23.6	62	21.7
>30	32	12.2	38	13.3
**Maternal Education**				
Less than high school/high school diploma/technical/ trade school	68	25.9	73	25.5
University degree	193	73.4	212	74.1
**Smoking during pregnancy**				
Yes	11	4.2	14	4.8
No	252	95.8	272	95.2
**Gestational age**				
<37 gestational week	13	4.9	21	7.3
≥37 gestational week	249	94.7	265	92.7
**Household Income**				
<$70,000	41	15.6	59	20.6
≥$70,000	222	84.4	225	78.7
**Marital Status**				
Married/cohabitating	256	97.3	279	97.6
Unmarried/single/ divorce/separated/widowed	5	1.9	5	1.7
Missing	2	0.8	2	0.7
**Race**				
White	227	86.3	246	86.0
Non-white	36	13.7	40	14.0
**Child birthweight (g)**				
<2500	12	4.6	16	5.6
≥2500	251	95.4	270	94.4

BMI = body mass index.

**Table 3:** Detection rates and distribution of maternal concentrations (µg/L) of phthalate metabolites and bisphenols in urine.

							Percentile						
Parent Compound	Abbreviation	Metabolite (µg/L)	Abbrev	LOD	% > LOD	GM	Minimum	5th	25th	50th	75th	95th	Maximum
**Phthalates**													
Di-methyl phthalate	DMP	Mono-methyl phthalate	MMP	0.10 µg/L	99.4	2.3	<LOD	0.4	1.3	2.4	4.2	9.8	98.7
Di-ethyl phthalate	DEP	Mono-ethyl phthalate	MEP	0.10 µg/L	100	50.4	0.1	5.2	17.7	45.4	144.2	651.2	22,520.6
Di-n-butyl phthalate	DBP	Mono-n-butyl phthalate	MBP	0.10 µg/L	99.1	16.2	<LOD	2.8	8.9	16.6	30.3	81.9	1129.7
Di-iso-butyl phthalate	DiBP	Mono-iso-butyl phthalate	MiBP	0.10 µg/L	98.9	9.4	<LOD	1.9	5.6	10.3	17.6	41.5	374.2
Di-(2-ethyl-hexyl) phthalate	DEHP	Mono-(2-ethyl-5-carboxypentyl) phthalate	MECPP	0.10 µg/L	100	14.2	0.4	2.7	7.8	14.9	26.4	68.3	341.6
Di-(2-ethyl-hexyl) phthalate	DEHP	Mono(2-ethyl-5-hydroxyhexyl) phthalate	MEHHP	0.10 µg/L	100	9.7	0.2	1.8	5.2	10.1	18.4	49.3	250.4
Di-(2-ethyl-hexyl) phthalate	DEHP	Mono(2-ethyl-5-oxohexyl) phthalate	MEOHP	0.10 µg/L	100	8.1	0.2	1.5	4.5	8.4	15.2	24.5	192.3
Di-(2-ethyl-hexyl) phthalate	DEHP	Mono(2-ethylhexyl) phthalate	MEHP	0.10 µg/L	99.1	3.2	<LOD	0.5	1.6	3.2	6.3	17.7	120.1
Benzylbutyl phthalate	BBzP	Monobenzyl phthalate	MBzP	0.10 µg/L	99.2	7.5	<LOD	1.1	3.5	8.1	16.1	54.0	515.5
Di-isononyl phthalate	DiNP	Monocarboxy-isooctyl phthalate	MCOP	0.10 µg/L	99.8	12.5	<LOD	1.5	5.1	11.8	27.4	143.9	1139.7
Di-isononyl phthalate	DiNP	Mono-isononyl phthalate	MNP	0.10 µg/L	99.8	4.1	<LOD	0.4	1.3	3.1	8.7	125.9	1612.5
Di-isodecyl phthalate	DiDP	Mononcarboxy-isononyl phthalate	MCNP	0.10 µg/L	91.0	1.1	<LOD	0.1	0.5	1.1	2.2	9.3	353.9
Di-cyclohexyl phthalate	DCHP	Mono-cyclohexyl phthalate	MCHP	0.10 µg/L	<50.0	0.1	<LOD	<LOD	<LOD	<LOD	0.1	0.9	29.9
Di-n-octyl phthalate	DOP	Mono-n-octyl phthalate	MOP	0.10 µg/L	<50.0	0.1	<LOD	<LOD	<LOD	<LOD	0.1	0.5	66.2
**Bisphenols**													
Bisphenol A	BPA	-	-	0.32 µg/mL	92.3	1.1	<LOD	<LOD	0.5	1.0	2.1	6.9	44.1
Bisphenol S	BPS	-	-	0.10 µg/mL	58.6	0.1	<LOD	<LOD	<LOD	0.12	0.3	1.1	243.1

GM: Geometric mean; LOD: Limit of detection.

**Table 4:** Prevalence and incidence of food allergies, eczema, rash, and asthma at 12, 24, and 36 months.

	N	Prevalence	N	Incidence
**Food Allergies**				
12 months	40	7.2	40	7.2
24 months	103	18.5	63	11.3
36 months	156	28.1	53	9.5
**Eczema**				
12 months	105	18.9	105	18.9
36 months	197	35.4	92	16.5
**Rash**				
12 months	189	33.9	189	33.9
36 months	340	61.1	151	27.1
**Asthma**				
12 months	2	0.4	2	0.4
24 months	11	1.9	9	1.6
36 months	33	5.7	22	3.9

**Table 5:** Associations between maternal urinary concentrations of phthalates, and food allergies, eczema, rash and asthma at 12, 24, and 36 months. Results are presented as adjusted odds ratios (AORs) with 95% confidence intervals (CIs).

	N	MMP	MEP	MBP	MiBP	MECPP	MEHHP	MEOHP	MEHP	MBzP	MCOP	MNP	MCNP	ΣDEHP
**Food Allergies**														
12 months	556	0.81 (0.54–1.23)	0.88 (0.68–1.13)	1.19 (0.82–1.72)	0.96 (0.62–1.48)	0.81 (0.51–1.27)	0.85 (0.56–1.30)	0.85 (0.54–1.34)	0.87 (0.62–1.23)	0.89 (0.62–1.28)	1.08 (0.82–1.42)	1.08 (0.89–1.31)	1.02 (0.84–1.24)	0.80 0.51–1.25)
24 months	556	0.82 (0.59–1.14)	1.02 (0.85–1.23)	0.97 (0.73–1.30)	1.00 (0.73–1.39)	1.09 (0.78–1.51)	0.93 (0.67–1.27)	0.98 (0.70–1.37)	0.91 (0.70–1.18)	**0.74 (0.55–1.00) ^†^**	0.95 (0.76–1.20)	0.89 (0.74–1.06)	1.03 (0.89–1.19)	0.95 (0.69–1.31)
36 months	556	1.17 (0.83–1.63)	0.97 (0.80–1.19)	0.81 (0.60–1.11)	0.91 (0.65–1.27)	0.82 (0.57–1.18)	0.86 (0.62–1.21)	0.84 (0.58–1.20)	1.12 (0.87–1.43)	1.22 (0.92–1.63)	1.10 (0.86–1.39)	1.05 (0.89–1.23)	0.94 (0.79–1.12)	0.98 (0.70–1.36)
**Eczema**														
12 months	556	**0.77 (0.58–1.02) ^†^**	0.90 (0.77–1.06)	0.98 (0.76–1.25)	**0.78 (0.59–1.02) ^†^**	1.09 (0.82–1.45)	1.07 (0.82–1.40)	1.01 (0.76–1.34)	1.04 (0.85–1.28)	**0.76 (0.60–0.97)** *	1.10 (0.91–1.33)	1.03 (0.90–1.18)	0.97 (0.85–1.11)	1.11 (0.85–1.44)
36 months	556	**1.29 (1.00–1.68)** *	0.90 (0.77–1.05)	0.89 (0.70–1.14)	0.88 (0.67–1.15)	1.13 (0.86–1.50)	1.09 (0.84–1.41)	1.06 (0.80–1.40)	1.11 (0.91–1.36)	1.02 (0.81–1.28)	**0.78 (0.64–0.96)** *	0.93 (0.8–1.07)	**1.11 (0.99–1.25) ^†^**	1.15 (0.89–1.49)
**Rash**														
12 months	556	0.88 (0.70–1.11)	0.94 (0.82–1.07)	1.07 (0.87–1.31)	0.96 (0.76–1.21)	1.14 (0.90–1.44)	1.09 (0.88–1.36)	1.05 (0.83–1.33)	1.07 (0.90–1.27)	0.89 (0.73–1.08)	**1.21 (1.03–1.41)** *	1.04 (0.93–1.16)	0.95 (0.85–1.07)	1.12 (0.90–1.40)
36 months	556	**1.35 (1.08–1.69)** *	0.98 (0.87–1.11)	1.02 (0.84–1.25)	1.01 (0.81–1.27)	1.02 (0.81–1.28)	1.05 (0.85–1.30)	1.02 (0.81–1.29)	**1.17 (0.98–1.39) ^†^**	1.12 (0.93–1.35)	**0.86 (0.73–1.01) ^†^**	0.91 (0.82–1.02)	1.03 (0.92–1.14)	1.16 (0.93–1.44)
**Asthma**														
12 months	556	0.18 (0.02–2.14)	1.29 (0.26–6.23)	2.10 (0.46–9.56)	0.56 (0.05–6.29)	0.89 (0.09–8.46)	0.99 (0.13–7.57)	1.13 (0.14–8.94)	0.60 (0.10–3.66)	0.25 (0.01–4.31)	0.81 (0.19–3.41)	0.81 (0.25–2.53)	0.64 (0.10–4.15)	0.33 (0.01–8.45)
24 months	556	**2.08 (0.99–4.39) ^†^**	1.11 (0.68–1.81)	1.11 (0.48–2.52)	1.84 (0.67–5.06)	1.45 (0.60–3.50)	1.60 (0.73–3.51)	1.51 (0.64–3.56)	0.93 (0.45–1.93)	0.75 (0.34–1.63)	0.99 (0.53–1.86)	1.01 (0.64–1.57)	0.97 (0.61–1.58)	1.34 (0.61–2.96)
36 months	556	1.01 (0.59–1.73)	**1.27 (0.97–1.67) ^†^**	1.12 (0.67–1.88)	1.17 (0.63–2.14)	1.13 (0.64–1.98)	1.08 (0.64–1.83)	1.10 (0.63–1.91)	0.82 (0.53–1.28)	1.30 (0.83–2.05)	0.69 (0.43–1.09)	0.78 (0.56–1.10)	0.76 (0.49–1.17)	1.03 (0.61–1.76)

Adjusted for maternal education, maternal age, maternal pre-pregnancy BMI, marital status, gestational age at birth, household income, parity, child sex, race, and creatinine. MMP = Mono-methyl phthalate; MEP = Monoethyl phthalate; MBP = Mono-n-butyl phthalate; MiBP = Mono-isobutyl phthalate; MECPP = Mono (2-ethyl-5-carboxypentyl) phthalate; MEHHP = Mono (2-ethyl-5-hydroxyhexyl) phthalate; MEOHP = Mono (2-ethyl-5-oxohexyl) phthalate; MEHP = Mono (2-ethylhexyl) phthalate; MBzP = Monobenzyl phthalate; MCOP = Monocarboxy-isooctyl phthalate; MNP = mono-isononyl phthalate; MCNP = Monocarboxy-isononyl phthalate.; ΣDEHP = sum of di(2-ethylhexyl) phthalates. Values in bold indicate statistical significance at *p* < 0.05 (*) or *p* < 0.10 (^†^).

**Table 6:** Associations between maternal urinary bisphenol concentrations and child food allergies, eczema, rash and asthma at 12, 24, and 36 months. Results are presented in adjusted odd ratios (AORs) and 95% confidence intervals (CIs).

	N	BPA	BPS
**Food Allergies**			
12 months	556	0.91 (0.63–1.31)	1.17 (0.87–1.58)
24 months	556	0.90 (0.67–1.22)	0.83 (0.61–1.11)
36 months	556	1.10 (0.82–1.45)	0.95 (0.71–1.27)
**Eczema**			
12 months	556	0.86 (0.67–1.10)	0.98 (0.79–1.22)
36 months	556	0.99 (0.79–1.24)	**0.72 (0.56–0.93)** *
**Rash**			
12 months	556	0.89 (0.73–1.08)	1.08 (0.91–1.30)
36 months	556	1.05 (0.87–1.26)	**0.81 (0.67–0.98)** *
**Asthma**			
12 months	556	0.68 (0.07–6.01)	**2.15 (0.88–5.23)** **^†^**
24 months	556	0.98 (0.46–2.09)	1.26 (0.68–2.35)
36 months	556	1.11 (0.72–1.72)	0.81 (0.49–1.34)

Adjusted for maternal education, maternal age, maternal pre-pregnancy BMI, marital status, gestational age at birth, household income, parity, child sex, race, and creatinine. BPA = Bisphenol A; BPS = Bisphenol S. Values in bold indicate statistical significance at *p* < 0.05 (*) or *p* < 0.10 (^†^).

**Table 7:** Associations between prenatal phthalate metabolite and bisphenol concentrations and child eczema and rash at 36 months of age estimated using double LASSO regression. Results are presented as adjusted odd ratios (AORs) with 95% confidence intervals (CIs).

Outcome	N	Exposure	Adjusted OR	*p* Value
**Eczema 36 months**	556			
		MMP	**1.42 (1.07–1.88)**	**0.01** *
		MEP	0.87 (0.73–1.03)	0.13
		MBP	0.91 (0.65–1.28)	0.61
		MiBP	0.90 (0.61–1.33)	0.61
		MECPP	**2.12 (0.95–4.71)**	**0.06** **^†^**
		MEHHP	2.80 (0.69–11.30)	0.14
		MEOHP	0.18 (0.03–1.07)	0.60
		MEHP	1.20 (0.85–1.69)	0.28
		MBzP	1.09 (0.84–1.41)	0.49
		MCOP	**0.67 (0.51–0.89)**	**0.006** *
		MNP	1.01 (0.84–1.19)	0.93
		MCNP	1.07 (0.93–1.22)	0.31
		BPA	0.99 (0.76–0.93)	0.99
		BPS	**0.73 (0.57–0.93)**	**0.01** *
**Rash 36 months**	556			
		MMP	**1.41 (1.11–1.81)**	**0.005** *
		MEP	0.95 (0.83–1.09)	0.52
		MBP	0.99 (0.75–1.31)	0.96
		MiBP	0.95 (0.70–1.28)	0.75
		MECPP	1.09 (0.53–2.25)	0.79
		MEHHP	2.69 (0.82–8.83)	0.10
		MEOHP	**0.23 (0.05–1.02)**	**0.05** **^†^**
		MEHP	**1.49 (1.11–2.00)**	**0.007** *
		MBzP	1.10 (0.88–1.36)	0.37
		MCOP	**0.82 (0.69–0.98)**	**0.03** *
		MNP	0.95 (0.81–1.10)	0.50
		MCNP	1.03 (0.91–1.16)	0.56
		BPA	1.04 (0.85–1.28)	0.64
		BPS	**0.79 (0.66–0.96)**	**0.01** *

Adjusted for maternal education, maternal age, maternal pre-pregnancy BMI, marital status, gestational age at birth, household income, parity, child sex, race, and creatinine. MMP = Mono-methyl phthalate; MEP = Monoethyl phthalate; MBP = Mono-n-butyl phthalate; MiBP = Mono-isobutyl phthalate; MECPP = Mono (2-ethyl-5-carboxypentyl) phthalate; MEHHP = Mono (2-ethyl-5-hydroxyhexyl) phthalate; MEOHP = Mono (2-ethyl-5-oxohexyl) phthalate; MEHP = Mono (2-ethylhexyl) phthalate; MBzP = Monobenzyl phthalate; MCOP = Monocarboxy-isooctyl phthalate; MNP = mono-isononyl phthalate; MCNP = Monocarboxy-isononyl phthalate; BPA = Bisphenol A; BPS = Bisphenol S. * *p* < 0.05; ^†^ *p* < 0.10. Values in bold indicate statistical significance at *p* < 0.05 (*) or *p* < 0.10 (^†^).

## Data Availability

Data is available from the APrON study (https://apronstudy.ca) upon request.

## Supplementary Materials

The following supporting information can be downloaded at https://www.mdpi.com/article/10.3390/ijerph22121875/s1, Figure S1: LASSO variable trace plots illustrating the coefficient trajectories of phthalate metabolites and bisphenols. The x-axis represents the sum of the absolute values of the penalized coefficients (the L1-norm). Each line shows the penalized coefficient path for one standardized variable in the model. The vertical red line marks the value of lambda (λ) selected through cross-validation; Figure S2: Univariate exposure–response functions of natural log (ln) transformed prenatal phthalate metabolites and bisphenols and eczema in children at 36 months of age. Associations between each analyte and maternal phthalate metabolites and bisphenols are plotted while fixing the other analytes at their 50th percentile (95% CI are shown in grey); Figure S3: Univariate exposure–response functions of natural log (ln) transformed prenatal phthalate metabolites and bisphenols and rash in children at 36 months of age. Associations between each analyte and maternal phthalate metabolites and bisphenols are plotted while fixing the other analytes at their 50th percentile (95% CI are shown in grey); Figure S4: Univariate exposure–response functions of natural log (ln) transformed prenatal phthalate metabolites and bisphenols and eczema in females at 36 months of age. Associations between each analyte and maternal phthalate metabolites and bisphenols are plotted while fixing the other analytes at their 50th percentile (95% CI are shown in grey); Figure S5: Univariate exposure–response functions of natural log (ln) transformed prenatal phthalate metabolites and bisphenols and rash in males at 36 months of age. Associations between each analyte and maternal phthalate metabolites and bisphenols are plotted while fixing the other analytes at their 50th percentile (95% CI are shown in grey); Figure S6: Overall mixture effect (95% CIs) of prenatal phthalate metabolites and bisphenols for eczema in females at 36 months of age using BKMR. This plot illustrated the change in predicted probability of eczema in females when all the ln-transformed analytes are at the respective quantile compared to when they are held at their median values; Figure S7: Overall mixture effect (95% CIs) of prenatal phthalate metabolites and bisphenols for rash in males at 36 months of age estimated using BKMR. This plot illustrated the change in predicted probability of rash in males when all the ln-transformed analytes are at the respective quantile compared to when they are held at their median values; Table S1: Spearman correlations between the environmental chemicals; Table S2: Adjusted sex-stratified associations between maternal urinary concentrations of each phthalate metabolite, and food allergies, eczema, rash and asthma. Results are presented as adjusted odd ratios (AORs) with 95% confidence interval (CIs); Table S3: Adjusted sex-stratified associations between maternal urinary concentrations of BPA and BPS, and food allergies, eczema, rash and asthma. Results are presented as adjusted odds ratios (AORs) with 95% confidence interval (CIs); Table S4: Posterior inclusion probabilities (PIPs) for prenatal phthalate metabolites and bisphenols in the Bayesian kernel machine regression (BKMR) model assessing eczema in children at 36 months of age; Table S5: Posterior inclusion probabilities (PIPs) for prenatal phthalate metabolites and bisphenols in the Bayesian kernel machine regression (BKMR) model assessing rash in children at 36 months of age; Table S6: Posterior inclusion probabilities (PIPs) for prenatal phthalate metabolites and bisphenols in the Bayesian kernel machine regression (BKMR) model assessing eczema in females at 36 months of age; Table S7: Posterior inclusion probabilities (PIPs) for prenatal phthalate metabolites and bisphenols in the Bayesian kernel machine regression (BKMR) model assessing rash in males at 36 months of age.

## Abbreviations

AOR	Adjusted odds ratio
APrON	Alberta Pregnancy Outcomes and Nutrition
BKMR	Bayesian kernel machine regression
BMI	Body mass index
BPA	Bisphenol A
BPS	Bisphenol S
CI	Confidence interval
DEHP	Di (2- ethylhexyl) phthalate
ΣDEHP	Molar sum of DEHP metabolites
GM	Geometric mean
HPLC-MS/MS	High-performance liquid chromatography-tandem mass spectrometry
EDCs	Endocrine disrupting chemicals
LASSO	Least absolute shrinkage and selection operator
LOD	Limit of detection
MBP	Mono-n-butyl phthalate
MBzP	Monobenzyl phthalate
MCHP	Mono-cyclohexyl phthalate
MCNP	Monocarboxy-isononyl phthalate
MCOP	Monocarboxy-isooctyl phthalate
MECPP	Mono (2-ethyl-5-carboxypentyl) phthalate
MEHHP	Mono (2-ethyl-5-hydroxyhexyl) phthalate
MEOHP	Mono (2-ethyl-5-oxohexyl) phthalate
MEHP	Mono (2-ethylhexyl) phthalate
MEP	Monoethyl phthalate
MiBP	Mono-isobutyl phthalate
MMP	Mono-methyl phthalate
MNP	Mono-isononyl phthalate
MOP	Mono-n-octyl phthalate
MRM	Multiple reaction monitoring
MSE	Mean squared error
OR	Odds ratio
PIPs	Posterior inclusion probabilities
PPARs	Peroxisome proliferator-activated receptors
Th1	Type 1 helper
Th2	Type 2 helper
T	Treg
WQS	Weighted quantile sums
