# Supplementary material for: Prenatal Exposure to Phthalates, Bisphenols and Their Mixtures and Early Childhood Allergic Conditions and Asthma: Results from the APrON Cohort
Source: Int J Environ Res Public Health. 2025 Dec 17;22(12):1875. doi: 10.3390/ijerph22121875 (PMC12732642; doi:10.3390/ijerph22121875)
Supplement: Supplementary file 1 [file ijerph-22-01875-s001.zip › ijerph-3957787-supplementary.pdf]

Supplemental Material

Prenatal Exposure to Phthalates, Bisphenols and their Mixtures and Early Childhood Allergic Conditions and Asthma: Results from the APrON Cohort

Emily Bartram,[1] Gillian England-Mason,[2,3] Jonathan W. Martin,[4] Amy M. MacDonald,[5] David W. Kinniburgh,[5,6] Deborah Dewey,[2,3,7,8*] Munawar Hussain Soomro,[2,3*]APrON Study Team

*These authors contributed equally to this work

Affiliations:

[1]Department of Biological Sciences, University of Calgary, Calgary, Canada

[2]Department of Pediatrics, Cumming School of Medicine, University of Calgary, Calgary, Canada

[3]Owerko Centre, Alberta Children's Hospital Research Institute, University of Calgary, Calgary, Canada

[4]Department of Environmental Science, Science for Life Laboratory, Stockholm University, Stockholm, Sweden

[5]Alberta Centre for Toxicology, University of Calgary, Calgary, Alberta, Canada

[6]Department of Laboratory Medicine and Pathology, University of Alberta, Edmonton, Alberta, Canada

[7]Department of Community Health Sciences, Cumming School of Medicine, University of Calgary, Calgary, Alberta, Canada

[8]Hotchkiss Brain Institute, University of Calgary, Calgary, Alberta, Canada.

Figures

1: LASSO variable trace plots illustrating the coefficient trajectories of phthalate metabolites and bisphenols. The x-axis represents the sum of the absolute values of the penalized coefficients (the L1-norm). Each line shows the penalized coefficient path for one standardized variable in the model. The vertical red line marks the value of lambda (ʎ) selected through cross-validation.

Figure S2. Univariate exposure-response functions of natural log (ln) transformed prenatal phthalate metabolites and bisphenols and eczema in children at 36 months of age. Associations between each analyte and maternal phthalate metabolites and bisphenols are plotted while fixing the other analytes at their 50th percentile (95% CI are shown in grey).

Figure S3. Univariate exposure-response functions of natural log (ln) transformed prenatal phthalate metabolites and bisphenols and rash in children at 36 months of age. Associations between each analyte and maternal phthalate metabolites and bisphenols are plotted while fixing the other analytes at their 50th percentile (95% CI are shown in grey).

Figure S4. Univariate exposure-response functions of natural log (ln) transformed prenatal phthalate metabolites and bisphenols and eczema in females at 36 months of age. Associations between each analyte and maternal phthalate metabolites and bisphenols are plotted while fixing the other analytes at their 50th percentile (95% CI are shown in grey).

Figure S5. Univariate exposure-response functions of natural log (ln) transformed prenatal phthalate metabolites and bisphenols and rash in males at 36 months of age. Associations between each analyte and maternal phthalate metabolites and bisphenols are plotted while fixing the other analytes at their 50th percentile (95% CI are shown in grey).

Figure S6. Overall mixture effect (95% CIs) of prenatal phthalate metabolites and bisphenols for eczema in females at 36 months of age using BKMR. This plot illustrated the change in predicted probability of eczema in females when all the ln-transformed analytes are at the respective quantile compared to when they are held at their median values.

Figure S7. Overall mixture effect (95% CIs) of prenatal phthalate metabolites and bisphenols for rash in males at 36 months of age estimated using BKMR. This plot illustrated the change in predicted probability of rash in males when all the ln-transformed analytes are at the respective quantile compared to when they are held at their median values.

Tables

Table S1. Spearman correlations between the environmental chemicals**.**

Table S2. Adjusted sex-stratified associations between maternal urinary concentrations of each phthalate metabolite, and food allergies, eczema, rash and asthma. Results are presented as adjusted odd ratios (AORs) with 95% confidence interval (CIs).

Table S3. Adjusted sex-stratified associations between maternal urinary concentrations of BPA and BPS, and food allergies, eczema, rash and asthma. Results are presented as adjusted odds ratios (AORs) with 95% confidence interval (CIs).

Table S4. Posterior inclusion probabilities (PIPs) for prenatal phthalate metabolites and bisphenols in the Bayesian kernel machine regression (BKMR) model assessing eczema in children at 36 months of age.

Table S5. Posterior inclusion probabilities (PIPs) for prenatal phthalate metabolites and bisphenols in the Bayesian kernel machine regression (BKMR) model assessing rash in children at 36 months of age.

Table S6. Posterior inclusion probabilities (PIPs) for prenatal phthalate metabolites and bisphenols in the Bayesian kernel machine regression (BKMR) model assessing eczema in females at 36 months of age.

Table S7. Posterior inclusion probabilities (PIPs) for prenatal phthalate metabolites and bisphenols in the Bayesian kernel machine regression (BKMR) model assessing rash in males at 36 months of age.

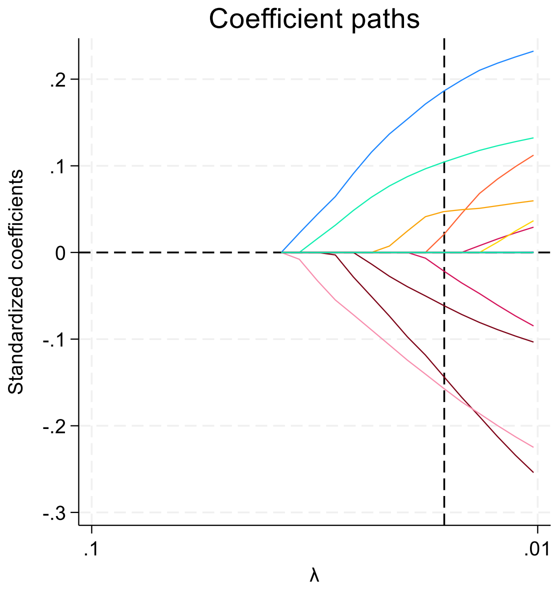

Figure S1-A: Eczema 36

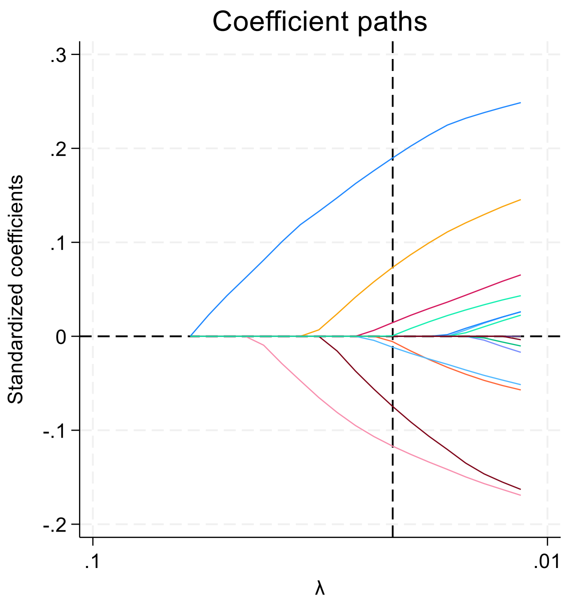

Figure S1-B: Rash 36

Figure S1: LASSO variable trace plots illustrating the coefficient trajectories of phthalate metabolites and bisphenols. The x-axis represents the sum of the absolute values of the penalized coefficients (the L1-norm). Each line shows the penalized coefficient path for one standardized variable in the model. The vertical red line marks the value of lambda (ʎ) selected through cross-validation.

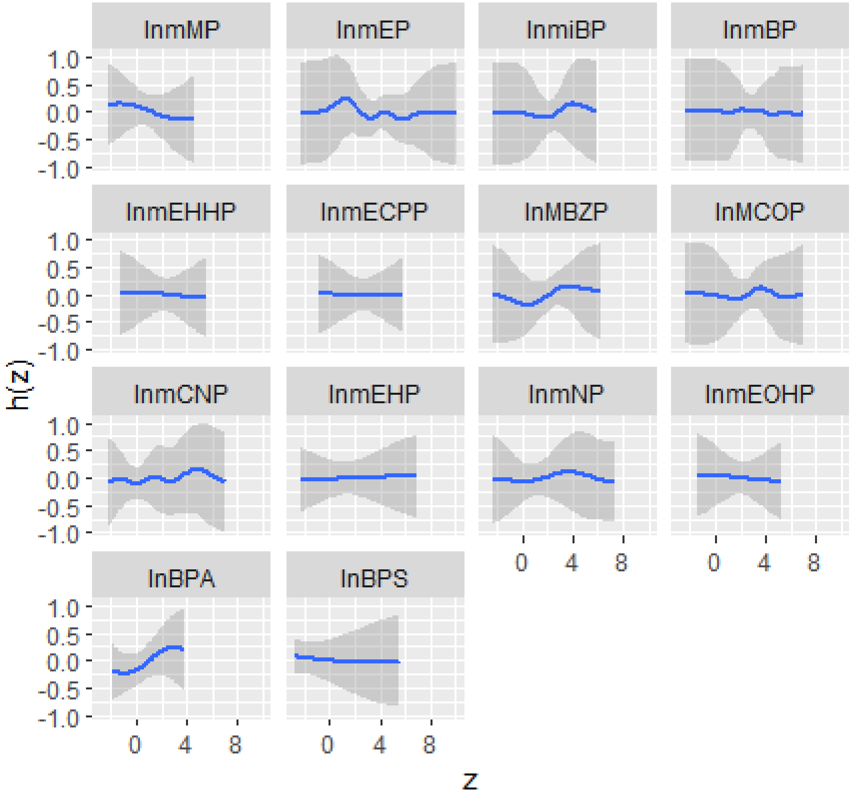

Figure S2. Univariate exposure-response functions of natural log (ln) transformed prenatal phthalate metabolites and bisphenols and eczema in children at 36 months of age. Associations between each analyte and maternal phthalate metabolites and bisphenols are plotted while fixing the other analytes at their 50th percentile (95% CI are shown in grey). Adjusted for maternal education, maternal age, maternal pre-pregnancy BMI, marital status, gestational age at birth, household income, parity, child sex, race, and creatinine. MMP = Mono-methyl phthalate; MEP = Monoethyl phthalate; MBP = Mono-n-butyl phthalate; MiBP = Mono-isobutyl phthalate; MECPP = Mono (2-ethyl-5-carboxypentyl) phthalate; MEHHP = Mono (2-ethyl-5-hydroxyhexyl) phthalate; MEOHP = Mono (2-ethyl-5-oxohexyl) phthalate; MEHP = Mono (2-ethylhexyl) phthalate; MBzP = Monobenzyl phthalate; MCOP = Monocarboxy-isooctyl phthalate; MNP = mono-isononyl phthalate; MCNP = Monocarboxy-isononyl phthalate; BPA = Bisphenol A; BPS = Bisphenol S.

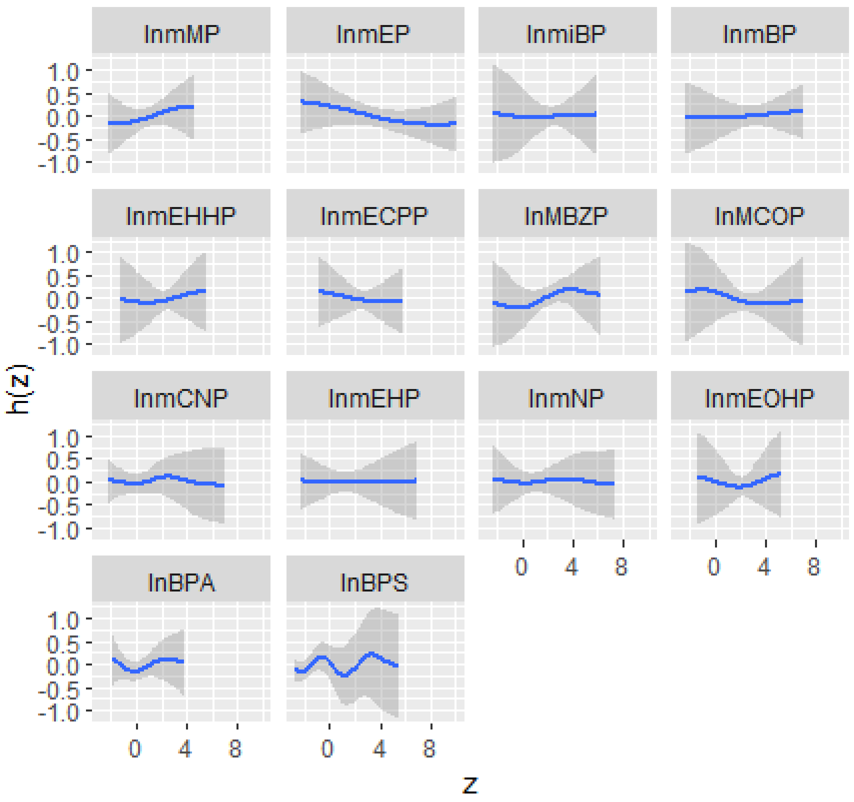

Figure S3. Univariate exposure-response functions of natural log (ln) transformed prenatal phthalate metabolites and bisphenols and rash in children at 36 months of age. Associations between each analyte and maternal phthalate metabolites and bisphenols are plotted while fixing the other analytes at their 50th percentile (95% CI are shown in grey). Adjusted for maternal education, maternal age, maternal pre-pregnancy BMI, marital status, gestational age at birth, household income, parity, child sex, race, and creatinine. MMP = Mono-methyl phthalate; MEP = Monoethyl phthalate; MBP = Mono-n-butyl phthalate; MiBP = Mono-isobutyl phthalate; MECPP = Mono (2-ethyl-5-carboxypentyl) phthalate; MEHHP = Mono (2-ethyl-5-hydroxyhexyl) phthalate; MEOHP = Mono (2-ethyl-5-oxohexyl) phthalate; MEHP = Mono (2-ethylhexyl) phthalate; MBzP = Monobenzyl phthalate; MCOP = Monocarboxy-isooctyl phthalate; MNP = mono-isononyl phthalate; MCNP = Monocarboxy-isononyl phthalate; BPA = Bisphenol A; BPS = Bisphenol S.

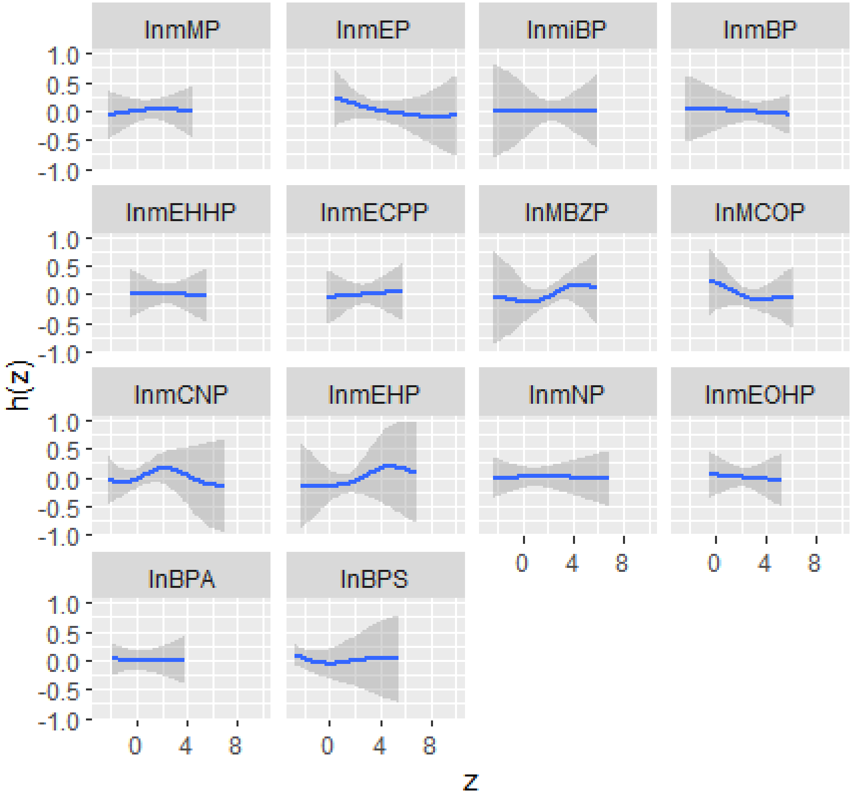

Figure S4. Univariate exposure-response functions of natural log (ln) transformed prenatal phthalate metabolites and bisphenols and eczema in females at 36 months of age. Associations between each analyte and maternal phthalate metabolites and bisphenols are plotted while fixing the other analytes at their 50th percentile (95% CI are shown in grey). Adjusted for maternal education, maternal age, maternal pre-pregnancy BMI, marital status, gestational age at birth, household income, parity, race, and creatinine. MMP = Mono-methyl phthalate; MEP = Monoethyl phthalate; MBP = Mono-n-butyl phthalate; MiBP = Mono-isobutyl phthalate; MECPP = Mono (2-ethyl-5-carboxypentyl) phthalate; MEHHP = Mono (2-ethyl-5-hydroxyhexyl) phthalate; MEOHP = Mono (2-ethyl-5-oxohexyl) phthalate; MEHP = Mono (2-ethylhexyl) phthalate; MBzP = Monobenzyl phthalate; MCOP = Monocarboxy-isooctyl phthalate; MNP = mono-isononyl phthalate; MCNP = Monocarboxy-isononyl phthalate; BPA = Bisphenol A; BPS = Bisphenol S.

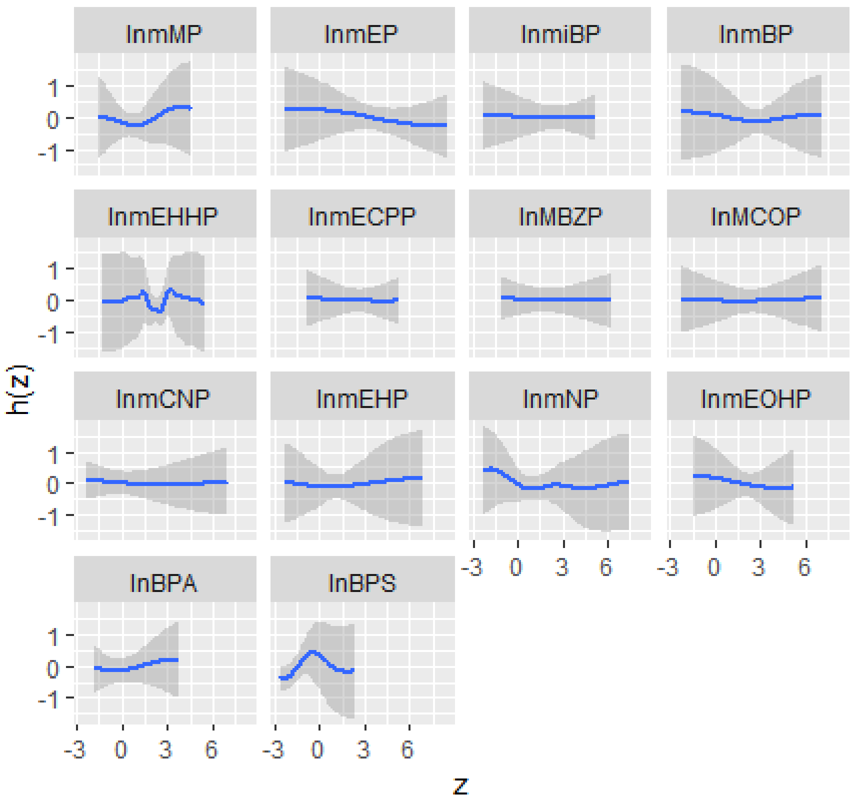

Figure S5. Univariate exposure-response functions of natural log (ln) transformed prenatal phthalate metabolites and bisphenols and rash in males at 36 months of age. Associations between each analyte and maternal phthalate metabolites and bisphenols are plotted while fixing the other analytes at their 50th percentile (95% CI are shown in grey). Adjusted for maternal education, maternal age, maternal pre-pregnancy BMI, marital status, gestational age at birth, household income, parity, race, and creatinine. MMP = Mono-methyl phthalate; MEP = Monoethyl phthalate; MBP = Mono-n-butyl phthalate; MiBP = Mono-isobutyl phthalate; MECPP = Mono (2-ethyl-5-carboxypentyl) phthalate; MEHHP = Mono (2-ethyl-5-hydroxyhexyl) phthalate; MEOHP = Mono (2-ethyl-5-oxohexyl) phthalate; MEHP = Mono (2-ethylhexyl) phthalate; MBzP = Monobenzyl phthalate; MCOP = Monocarboxy-isooctyl phthalate; MNP = mono-isononyl phthalate; MCNP = Monocarboxy-isononyl phthalate; BPA = Bisphenol A; BPS = Bisphenol S.

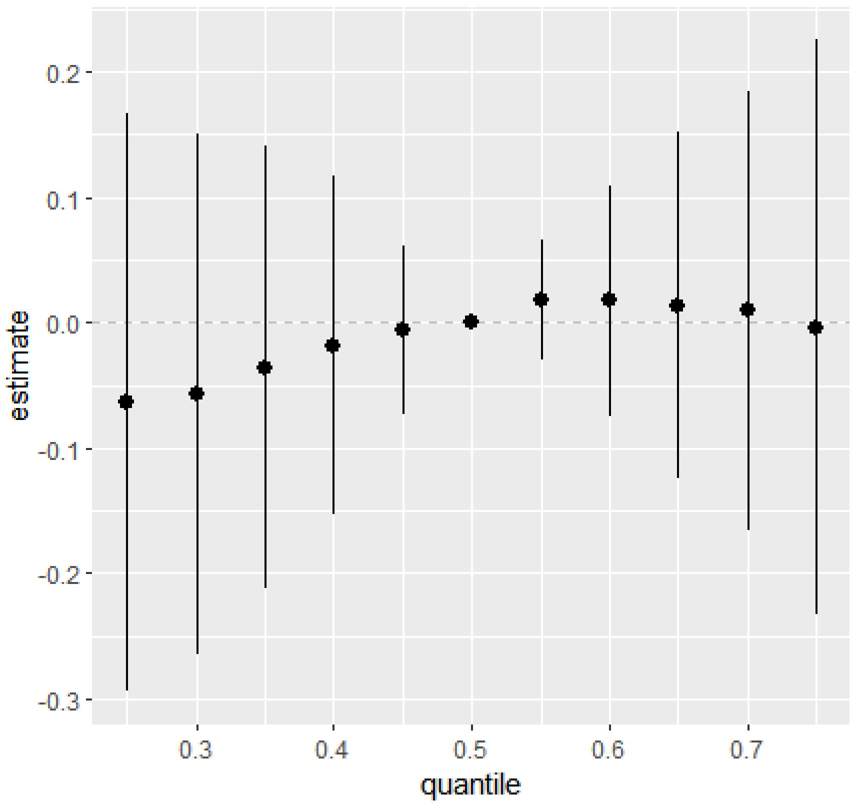

Figure S6. Overall mixture effect (95% CIs) of prenatal phthalate metabolites and bisphenols for eczema in females at 36 months of age using BKMR. This plot illustrated the change in predicted probability of eczema in females when all the ln-transformed analytes are at the respective quantile compared to when they are held at their median values. Adjusted for maternal education, maternal age, maternal pre-pregnancy BMI, marital status, gestational age at birth, household income, parity, race, and creatinine.

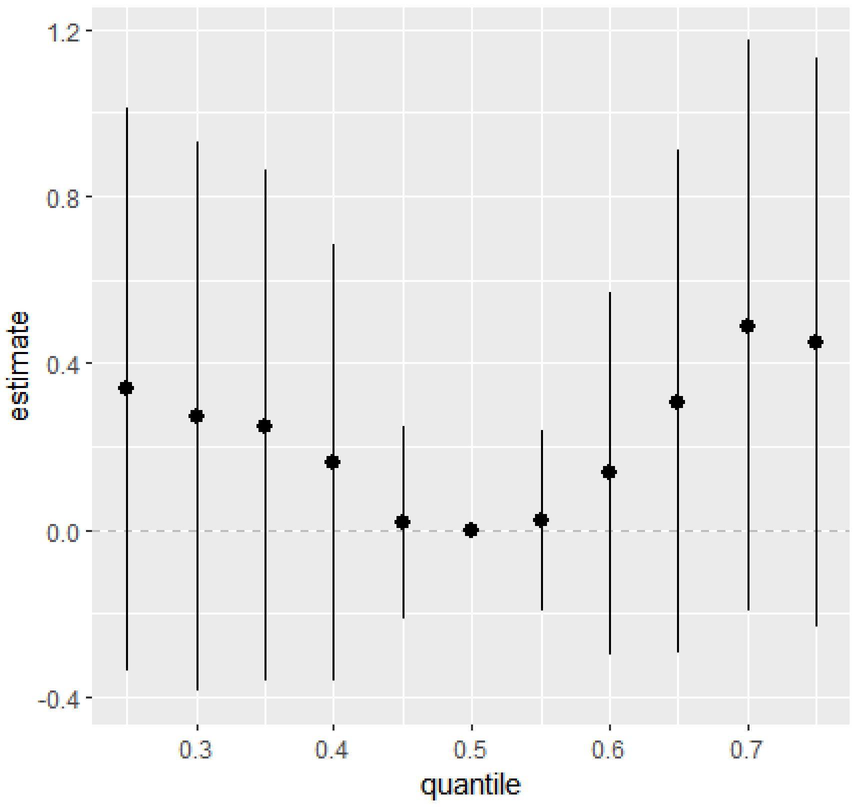

Figure S7. Overall mixture effect (95% CIs) of prenatal phthalate metabolites and bisphenols for rash in males at 36 months of age estimated using BKMR. This plot illustrated the change in predicted probability of rash in males when all the ln-transformed analytes are at the respective quantile compared to when they are held at their median values. Adjusted for maternal education, maternal age, maternal pre-pregnancy BMI, marital status, gestational age at birth, household income, parity, race, and creatinine.

Table S1. Spearman correlations between the environmental chemicals.

	MMP	MEP	MBP	MiBP	MECPP	MEHHP	MEOHP	MEHP	MBzP	MCOP	MNP	MCNP	BPA
**MEP**	0.33*												
**MBP**	0.50*	0.37*											
**MiBP**	0.54*	0.42*	0.74*										
**MECPP**	0.50*	0.37*	0.61*	0.65*									
**MEHHP**	0.51*	0.34*	0.62*	0.63*	0.94*								
**MEOHP**	0.52*	0.37*	0.66*	0.67*	0.95*	0.98*							
**MEHP**	0.42*	0.30*	0.51*	0.52*	0.80*	0.84*	0.84*						
**MBzP**	0.43*	0.32*	0.64*	0.58*	0.48*	0.48*	0.50*	0.41*					
**MCOP**	0.38*	0.26*	0.46*	0.47*	0.61*	0.58*	0.55*	0.50*	0.40*				
**MNP**	0.27*	0.14*	0.31*	0.34*	0.50*	0.52*	0.50*	0.84*	0.32*	0.73*			
**MCNP**	0.31*	0.18*	0.32*	0.40*	0.45*	0.39*	0.40*	0.36*	0.30*	0.50*	0.41*		
**BPA**	0.39*	0.21*	0.47*	0.50*	0.47*	0.49*	0.50*	0.41*	0.41*	0.38*	0.32*	0.30*	
**BPS**	0.27*	0.18*	0.28*	0.34*	0.33*	0.35*	0.36*	0.30*	0.25*	0.27*	0.27*	0.18*	0.27*

MMP = Mono-methyl phthalate; MEP = Monoethyl phthalate; MBP = Mono-n-butyl phthalate; MiBP = Mono-isobutyl phthalate; MECPP = Mono (2-ethyl-5-carboxypentyl) phthalate; MEHHP = Mono (2-ethyl-5-hydroxyhexyl) phthalate; MEOHP = Mono (2-ethyl-5-oxohexyl) phthalate; MEHP = Mono (2-ethylhexyl) phthalate; MBzP = Monobenzyl phthalate; MCOP = Monocarboxy-isooctyl phthalate; MNP = mono-isononyl phthalate; MCNP = Monocarboxy-isononyl phthalate; BPA= Bisphenol A; BPS = Bisphenol S

*$p < 0.05$

Table S2. Adjusted sex-stratified associations between maternal urinary concentrations of each phthalate metabolite, and food allergies, eczema, rash and asthma. Results are presented as adjusted odd ratios (AORs) with 95% confidence interval (CIs).

		**MMP**	**MEP**	**MBP**	**MiBP**	**MECPP**	**MEHHP**	**MEOHP**	**MEHP**	**MBzP**	**MCOP**	**MNP**	**MCNP**	**ΣDEHP**
**Food Allergies**														
12 months	**Females**	0.89 (0.46-1.73)	0.87 (0.58-1.29)	**1.80 (0.96-3.39)**†	1.07 (0.50-2.27)	0.86 (0.40-1.82)	0.94 (0.50-1.79)	1.01 (0.52-1.98)	0.96 (0.57-1.63)	0.99 (0.55-1.77)	0.83 (0.48-1.43)	0.77 (0.51-1.18)	1.03 (0.76-1.40)	0.92 (0.46-1.84)
	**Males**	0.74 (0.41-1.33)	0.90 (0.64-1.28)	0.99 (0.61-1.61)	0.86 (0.50-1.47)	0.80 (0.44-1.46)	0.81 (0.45-1.45)	0.75 (0.39-1.42)	0.80 (0.49-1.31)	0.89 (0.54-1.47)	1.20 (0.85-1.69)	**1.24 (0.97-1.60)**†	1.01 (0.78-1.31)	0.75 (0.41-1.38)
24 months	**Females**	**0.58 (0.36-0.93)***	1.08 (0.83-1.41)	1.07 (0.67-1.69)	0.93 (0.58-1.49)	0.99 (0.61-1.61)	0.84 (0.53-1.32)	.93 (0.58-1.50)	0.91 (0.64-1.30)	0.73 (0.48-1.11)	0.86 (0.60-1.23)	0.84 (0.65-1.09)	0.99 (0.80-1.23)	0.87 (0.55-1.39)
	**Males**	1.09 (0.69-1.74)	0.94 (0.72-1.22)	0.89 (0.60-1.33)	1.05 (0.65-1.70)	1.22 (0.76-1.98)	1.04 (0.65-1.65)	1.05 (0.64-1.71)	0.92 (0.63-1.34)	0.74 (0.48-1.15)	1.4 (0.76-1.42)	0.92 (0.72-1.16)	1.03 (0.83-1.27)	1.04 (0.65-1.66)
36 months	**Females**	0.94 (0.56-1.58)	1.13 (0.84-1.51)	1.05 (0.63-1.75)	1.03 (0.60-1.77)	0.95 (0.57-1.59)	1.05 (0.66-1.64)	1.03 (0.63-1.67)	1.31 (0.94-1.83)	**1.60 (1.06-2.42)***	0.95 (0.64-1.42)	0.98 (0.75-1.27)	**0.70 (0.47-1.05)**†	1.34 (0.86-2.08)
	**Males**	1.35 (0.85-2.14)	0.86 (0.66-1.13)	**0.68 (0.45-1.02)**†	0.80 (0.52-1.23)	0.69 (0.41-1.16)	0.66 (0.40-1.08)	0.64 (0.37-1.09)	0.91 (0.62-1.33)	0.96 (0.64-1.44)	1.20 (0.88-1.63)	1.11 (0.90-1.39)	1.06 (0.87-1.29)	0.66 (0.40-1.10)
**Eczema**														
12 months	**Females**	0.73 (0.48-1.12) 0.16	**0.69 (0.52-0.92)***	0.95 (0.63-1.45) 0.84	**0.62 (0.41-0.95)***	1.12 (0.73-1.72) 0.59	1.02 (0.68-1.51) 0.91	0.91 (0.59-1.41) 0.69	0.82 (0.58-1.16) 0.28	0.88 (0.61-1. Σ) 0.48	0.94 (0.68-1.30) 0.73	0.97 (0.77-1.21) 0.81	0.96 (0.76-1.21) 0.74	0.96 (0.64-1.46) 0.87
	**Males**	0.79 (0.53-1.17) 0.25	1.03 (0.84-1.28) 0.71	0.98 (0.72-1.34) 0.93	0.92 (0.64-1.33) 0.68	1.09 (0.74-1.62) 0.64	1.14 (0.79-1.64) 0.48	1.12 (0.76-1.66) 0.55	1.27 (0.95-1.70) 0.10	**0.66 (0.46-0.92)***	**1.23 (0.96-1.57)**†	1.07 (0.90-1.27) 0.38	0.97 (0.82-1.15) 0.79	1.24 (0.87-1.79) 0.22
36 months	**Females**	1.09 (0.74-1.61) 0.64	0.91 (0.72-1.16) 0.48	0.89 (0.59-1.34) 0.59	0.89 (0.58-1.37) 0.61	0.97 (0.63-1.51) 0.92	0.92 (0.62-1.37) 0.70	0.92 (0.60-1.41) 0.71	1.19 (0.89-1.60) 0.22	**1.30 (1.00-1.69)***	**0.61 (0.43-0.87)***	0.97 (0.79-1.19) 0.78	**1.25 (1.05-1.50)***	1.15 (0.8-1.69) 0.47
	**Males**	**1.47 (1.02-2.12)***	0.87 (0.70-1.07) 0.20	0.88 (0.64-1.21) 0.44	0.86 (0.60-1.25) 0.44	1.29 (0.88-1.89) 0.17	1.28 (0.89-1.84) 0.17	1.22 (0.83-1.79) 0.29	1.07 (0.81-1.42) 0.59	0.80 (0.57-1.11) 0.18	0.89 (0.69-1.16) 0.41	0.88 (0.73-1.07) 0.22	1.00 (0.84-1.19) 0.94	1.19 (0.83-1.70) 0.32
**Rash**														
12 months	**Females**	**0.71 (0.50-1.01)**†	**0.77 (0.61-0.95)***	1.12 (0.80-1.57)	0.84 (0.59-1.20)	1.22 (0.85-1.74)	1.09 (0.79-1.51)	1.03 (0.73-1.46)	0.89 (0.68-1.17)	1.07 (0.80-1.41)	1.04 (0.80-1.35)	0.97 0.81-1.16)	0.87 (0.71-1.06)	1.03 (0.74-1.44)
	**Males**	1.00 (0.74-1.37)	1.08 (0.91-1.29)	1.04 (0.80-1.35)	1.05 (0.77-1.43)	1.09 (0.79-1.51)	1.11 (0.81-1.51)	1.09 (0.78-1.50)	**1.25 (0.98-1.61)**†	**0.72 (0.55-0.96)***	**1.34 (1.08-1.66)***	1.09 (0.94-1.26)	1.01 (0.87-1.16)	1.22 (0.90-1.66)
36 months	**Females**	1.22 (0.88-1.70)	1.09 (0.90-1.32)	1.10 (0.80-1.52)	1.06 (0.74-1.51)	1.02 (0.72-1.43)	1.06 (0.78-1.45)	1.04.74-1.45)	1.17 (0.92-1.50)	1.18 (0.90-1.54)	0.83 (0.64-1.06)	0.99 (0.83-1.16)	**1.14 (0.97-1.33)**†	1.15 (0.84-1.58)

	**Males**	**1.45 (1.06-1.98)***	0.91 (0.76-1.08)	0.98 (0.75-1.27)	0.98 (0.72-1.34)	1.01 (0.73-1.38)	1.03 (0.76-1.39)	1.00 (0.72-1.38)	1.15 (0.91-1.46)	1.06 (0.81-1.37)	0.87 (0.71-1.07)	**0.85 (0.73-1.00)†**	0.96 (0.83-1.11)	1.15 (0.85-1.56)
**Asthma**														
12 months	**Females**	-	-	-	-	-	-	-	-	-	-	-	-	-
	**Males**	0.16 (0.01-1.63)	1.30 (0.27-6.28)	2.12 (0.46-9.62) 0.32	0.43 (0.02-8.01)	0.91 (0.09-8.56) 0.93	1.02 (0.13-7.63)	1.00 (0.11-9.06)	0.62 (0.11-3.48)	0.26 (0.01-4.38)	0.81 (0.19-3.41)	0.81 (0.25-2.54)	0.63 (0.09-4.22)	0.35 (0.01-8.17)
24 months	**Females**	1.98 (0.54-7.27)	1.20 (0.47-3.03)	3.65 (0.67-19.75) 0.13	**9.88 (0.79-122.57)†**	**4.17 (0.79-22.04)†**	**8.87 (0.98-79.75)†**	**5.97 (0.98-36.31)†**	1.80 (0.78-4.13)	0.87 (0.23-3.24)	0.84 (0.26-2.71)	1.04 (0.49-2.22)	0.75 (0.21-2.64)	**3.79 (0.91-15.78)†**
	**Males**	**2.30 (0.87-6.07)†**	1.16 (0.62-2.19) 0.63	0.75 (0.28-2.05)	1.22 (0.35-4.20)	0.94 (0.27-3.19)	0.89 (0.27-2.96)	0.88 (0.25-3.08)	0.57 (0.22-1.49)	0.58 (0.21-1.58)	1.04 (0.50-2.17)	0.97 (0.56-1.68)	1.02 (0.60-1.74)	0.83 (0.24-2.86)
36 months	**Females**	0.59 (0.21-1.66)	1.01 (0.60-1.72) 0.94	0.93 (0.32-2.68)	0.86 (0.30-2.48)	0.76 (0.22-2.63)	0.66 (0.22-1.97)	0.61 (0.18-2.07)	**0.43 (0.16-1.14)†**	**1.85 (0.96-3.58)†**	**0.23 (0.06-0.90)***	**0.38 (0.15-0.91)***	0.60 (0.24-1.48)	0.59 (0.16-2.07)
	**Males**	1.26 (0.66-2.39)	**1.33 (0.94-1.88)†**	1.19 (0.64-2.20)	1.25 (0.56-2.76)	1.29 (0.65-2.52)	1.31 (0.71-2.44)	1.35 (0.70-2.58)	0.98 (0.61-1.58)	0.97 (0.52-1.81)	0.88 (0.52-1.47)	0.91 (0.64-1.31)	0.77 (0.46-1.29)	1.12 (0.61-2.03)

Adjusted for maternal education, maternal age, maternal pre-pregnancy BMI, marital status, gestational age at birth, household income, parity, child sex, race, and creatinine.

MMP = Mono-methyl phthalate; MEP = Monoethyl phthalate; MBP = Mono-n-butyl phthalate; MiBP = Mono-isobutyl phthalate; MECPP = Mono (2-ethyl-5-carboxypentyl) phthalate; MEHHP = Mono (2-ethyl-5-hydroxyhexyl) phthalate; MEOHP = Mono (2-ethyl-5-oxohexyl) phthalate; MEHP = Mono (2-ethylhexyl) phthalate; DEHP = di(2- ethylhexyl) phthalate; MBzP = Monobenzyl phthalate; MCOP = Monocarboxy-isooctyl phthalate; MNP = mono-isononyl phthalate; MCNP = Monocarboxy-isononyl phthalate.

Values in bold indicate statistical significance at $p < 0.05$ (*) or $p < 0.10$ (†).

Table S3. Adjusted sex-stratified associations between maternal urinary concentrations of BPA and BPS, and food allergies, eczema, rash and asthma. Results are presented as adjusted odds ratios (AORs) with 95% confidence interval (CIs).

		BPA	BPS
**Food Allergies**			
12 months	**Females**	0.94 (0.55-1.61)	1.24 (0.85-1.82)
	**Males**	0.88 (0.53-1.45)	1.08 (0.66-1.77)
24 months	**Females**	1.02 (0.67-1.55)	**0.64 (0.39-1.04)**†
	**Males**	0.80 (0.52-1.24)	1.07 (0.72-1.60) 0.71
36 months	**Females**	1.17 (0.79-1.76)	1.12 (0.79-1.60) 0.51
	**Males**	0.98 (0.65-1.48)	**0.62(0.36-0.98)***
**Eczema**			
12 months	**Females**	0.81 (0.56-1.18)	0.91 (0.65-1.28)
	**Males**	0.88 (0.63-1.23)	1.09 (0.79-1.50)
36 months	**Females**	0.94 (0.67-1.32)	**0.70 (0.48-1.02)**†
	**Males**	1.02 (0.75-1.39)	0.79 (0.55-1.13)
**Rash**			
12 months	**Females**	0.81 (0.59-1.09)	1.06 (0.83-1.37)
	**Males**	0.95 (0.73-1.24)	1.18 (0.91-1.54)
36 months	**Females**	0.94 (0.72-1.24)	0.82 (0.62-1.08)
	**Males**	1.12 (0.87-1.45)	0.83 (0.63-1.09)
**Asthma**			
12 months	**Females**	-	-
	**Males**	0.75 (0.11-5.12)	**3.24 (0.97-10.81)**†
24 months	**Females**	0.88 (0.21-3.68)	1.70 (0.64-4.53)
	**Males**	1.00 (0.40-2.49)	1.14 (0.47-2.78)
36 months	**Females**	1.43 (0.69-2.93)	0.40 (0.11-1.41)
	**Males**	0.94 (0.52-1.70)	1.01 (0.55-1.84)

Adjusted for maternal education, maternal age, maternal pre-pregnancy BMI, marital status, gestational age at birth, household income, parity, child sex, race, and creatinine. BPA= Bisphenol A; BPS = Bisphenol S. Values in bold indicate statistical significance at $p < 0.05$ (*) or $p < 0.10$ (†).

Table S4. Posterior inclusion probabilities (PIPs) for prenatal phthalate metabolites and bisphenols in the Bayesian kernel machine regression (BKMR) model assessing eczema in children at 36 months of age.

Analyte	Group PIP	Conditional PIP
**MMP**	0.47	0.18
**MEP**	0.47	0.02
**MBP**	0.47	0.05
**MiBP**	0.47	0.05
**MECPP**	0.47	0.11
**MEHHP**	0.47	0.09
**MEOHP**	0.47	0.10
**MEHP**	0.47	0.07
**MBzP**	0.47	0.04
**MCOP**	0.47	0.15
**MNP**	0.47	0.01
**MCNP**	0.47	0.06
**BPA**	**0.69**	0.12
**BPS**	**0.69**	**0.87**

Adjusted for maternal education, maternal age, maternal pre-pregnancy BMI, marital status, gestational age at birth, household income, parity, child sex, race, and creatinine. MMP = Mono-methyl phthalate; MEP = Monoethyl phthalate; MBP = Mono-n-butyl phthalate; MiBP = Mono-isobutyl phthalate; MECPP = Mono (2-ethyl-5-carboxypentyl) phthalate; MEHHP = Mono (2-ethyl-5-hydroxyhexyl) phthalate; MEOHP = Mono (2-ethyl-5-oxohexyl) phthalate; MEHP = Mono (2-ethylhexyl) phthalate; MBzP = Monobenzyl phthalate; MCOP = Monocarboxy-isooctyl phthalate; MNP = mono-isononyl phthalate; MCNP = Monocarboxy-isononyl phthalate; BPA = Bisphenol A; BPS = Bisphenol S. Values in bold indicate PIP ≥ 0.5 (i.e., meaningful inclusion probabilities)

Table S5. Posterior inclusion probabilities (PIPs) for prenatal phthalate metabolites and bisphenols in the Bayesian kernel machine regression (BKMR) model assessing rash in children at 36 months of age.

Analyte	Group PIP	Conditional PIP
**MMP**	**0.53**	**0.47**
**MEP**	**0.53**	0.004
**MBP**	**0.53**	0.02
**MIBP**	**0.53**	0.03
**MECPP**	**0.53**	0.04
**MEHHP**	**0.53**	0.03
**MEOHP**	**0.53**	0.07
**MEHP**	**0.53**	0.12
**MBzP**	**0.53**	0.04
**MCOP**	**0.53**	0.11
**MNP**	**0.53**	0.01
**MCNP**	**0.53**	0.005
**BPA**	**0.65**	0.11
**BPS**	**0.65**	**0.88**

Adjusted for maternal education, maternal age, maternal pre-pregnancy BMI, marital status, gestational age at birth, household income, parity, child sex, race, and creatinine. MMP = Mono-methyl phthalate; MEP = Monoethyl phthalate; MBP = Mono-n-butyl phthalate; MiBP = Mono-isobutyl phthalate; MECPP = Mono (2-ethyl-5-carboxypentyl) phthalate; MEHHP = Mono (2-ethyl-5-hydroxyhexyl) phthalate; MEOHP = Mono (2-ethyl-5-oxohexyl) phthalate; MEHP = Mono (2-ethylhexyl) phthalate; MBzP = Monobenzyl phthalate; MCOP = Monocarboxy-isooctyl phthalate; MNP = mono-isononyl phthalate; MCNP = Monocarboxy-isononyl phthalate; BPA = Bisphenol A; BPS = Bisphenol S. Values in bold indicate PIPs ≥ 0.5 (i.e., meaningful inclusion probabilities)

Table S6. Posterior inclusion probabilities (PIPs) for prenatal phthalate metabolites and bisphenols in the Bayesian kernel machine regression (BKMR) model assessing eczema in females at 36 months of age.

Analyte	Group PIP	Conditional PIP
**MMP**	**0.64**	0.05
**MEP**	**0.64**	0.01
**MBP**	**0.64**	0.04
**MIBP**	**0.64**	0.05
**MECPP**	**0.64**	0.03
**MEHHP**	**0.64**	0.03
**MEOHP**	**0.64**	0.04
**MEHP**	**0.64**	0.03
**MBzP**	**0.64**	0.12
**MCOP**	**0.64**	0.28
**MNP**	**0.64**	0.005
**MCNP**	**0.64**	0.25
**BPA**	0.49	0.27
**BPS**	0.49	**0.72**

Adjusted for maternal education, maternal age, maternal pre-pregnancy BMI, marital status, gestational age at birth, household income, parity, child sex, race, and creatinine. MMP = Mono-methyl phthalate; MEP = Monoethyl phthalate; MBP = Mono-n-butyl phthalate; MiBP = Mono-isobutyl phthalate; MECPP = Mono (2-ethyl-5-carboxypentyl) phthalate; MEHHP = Mono (2-ethyl-5-hydroxyhexyl) phthalate; MEOHP = Mono (2-ethyl-5-oxohexyl) phthalate; MEHP = Mono (2-ethylhexyl) phthalate; MBzP = Monobenzyl phthalate; MCOP = Monocarboxy-isooctyl phthalate; MNP = mono-isononyl phthalate; MCNP = Monocarboxy-isononyl phthalate; BPA = Bisphenol A; BPS = Bisphenol S. Values in bold indicate PIP ≥ 0.5 (i.e., meaningful inclusion probabilities)

Table S7. Posterior inclusion probabilities (PIPs) for prenatal phthalate metabolites and bisphenols in the Bayesian kernel machine regression (BKMR) model assessing rash in males at 36 months of age.

Analyte	Group PIP	Conditional PIP
**MMP**	0.40	0.30
**MEP**	0.40	0.08
**MBP**	0.40	0.03
**MiBP**	0.40	0.06
**MECPP**	0.40	0.05
**MEHHP**	0.40	0.10
**MEOHP**	0.40	0.07
**MEHP**	0.40	0.05
**MBzP**	0.40	0.04
**MCOP**	0.40	0.08
**MNP**	0.40	0.06
**MCNP**	0.40	0.01
**BPA**	**0.68**	0.19
**BPS**	**0.68**	**0.80**

Adjusted for maternal education, maternal age, maternal pre-pregnancy BMI, marital status, gestational age at birth, household income, parity, race, and creatinine. MMP = Mono-methyl phthalate; MEP = Monoethyl phthalate; MBP = Mono-n-butyl phthalate; MiBP = Mono-isobutyl phthalate; MECPP = Mono (2-ethyl-5-carboxypentyl) phthalate; MEHHP = Mono (2-ethyl-5-hydroxyhexyl) phthalate; MEOHP = Mono (2-ethyl-5-oxohexyl) phthalate; MEHP = Mono (2-ethylhexyl) phthalate; MBzP = Monobenzyl phthalate; MCOP = Monocarboxy-isooctyl phthalate; MNP = mono-isononyl phthalate; MCNP = Monocarboxy-isononyl phthalate; BPA = Bisphenol A; BPS = Bisphenol S. Values in bold indicate PIP ≥ 0.5 (i.e., meaningful inclusion probabilities
